# Author Correction: IRF3 prevents colorectal tumorigenesis via inhibiting the nuclear translocation of β-catenin

**DOI:** 10.1038/s41467-023-43761-7

**Published:** 2023-11-29

**Authors:** Miao Tian, Xiumei Wang, Jihong Sun, Wenlong Lin, Lumin Chen, Shengduo Liu, Ximei Wu, Liyun Shi, Pinglong Xu, Xiujun Cai, Xiaojian Wang

**Affiliations:** 1 Institute of Immunology and Bone Marrow Transplantation Center, The First Affiliated Hospital, School of Medicine, Zhejiang University, 310003 Hangzhou, China; 2 Department of Radiology, Sir Run Run Shaw Hospital, School of Medicine, Zhejiang University, 310016 Hangzhou, China; 3 The MOE Key Laboratory of Biosystems Homeostasis & Protection and Innovation Center for Cell Signaling Network, Life Sciences Institute, Zhejiang University, 310058 Hangzhou, China; 4 Department of Pharmacology, School of Medicine, Zhejiang University, 310058 Hangzhou, Zhejiang China; 5 Department of Immunology and Medical Microbiology, Nanjing University of Chinese Medicine, 210046 Nanjing, China; 6 Department of General Surgery, Innovation Center for Minimally Invasive Techniques and Devices, Sir Run Run Shaw Hospital, Zhejiang University School of Medicine, 310016 Hangzhou, China

**Keywords:** Gynaecological cancer, Cell biology, Gastroenterology

Correction to: *Nature Communications* 10.1038/s41467-020-19627-7,published online 13 November 2020

The original version of this Article contained errors in Figure 2e in which the immunostaining image of D90-paracancerous tissue from IRF3^+/+^ →IRF3^-/-^ chimera was the duplicated image of D90-tumour image of the IRF3^-/-^ mouse from Figure 2a and the immunostaining images of D90-paracancerous tissue from IRF3^-/-^ → IRF3^+/+^ chimera were inadvertently duplicated from the image of D90 paracancerous tissue from IRF3^+/+^ → IRF3^+/+^ chimera. The IRF3^+/+^ → IRF3^+/+^ D90-tumor tissue image and the IRF3^-/-^ → IRF3^+/+^ D90-tumor tissue image were inadvertently switched during the assembly.

These errors have been corrected in the PDF and HTML versions of the Article.

The raw data of the two independent experiments of Figures 2a and 2e are available as source data. These corrections will not affect any interpretation or conclusion of the experiment.

### Supplementary information

Raw Data

