# Supplementary material for: IRF3 prevents colorectal tumorigenesis via inhibiting the nuclear translocation of β-catenin
Source: Nat Commun. 2020 Nov 13;11:5762. doi: 10.1038/s41467-020-19627-7 (PMC7666182; doi:10.1038/s41467-020-19627-7)
Supplement: Supplementary file 1 — Supplementary Information [file 41467_2020_19627_MOESM1_ESM.pdf]

# IRF3 prevents colorectal tumorigenesis via inhibiting the nuclear translocation of β-catenin

**1. Supplementary Figures and legends**

Supplementary Figure 1, related to Figure 1; Supplementary Figure 2, related to Figure 2;
Supplementary Figure 3, related to Figure 3; Supplementary Figure 4, related to Figure 4;
Supplementary Figure 5, related to Figure 5; Supplementary Figure 6, related to Figure 6;
Supplementary Figure 7, related to Figure 7; Supplementary Figure 8

**2. Supplementary methods**

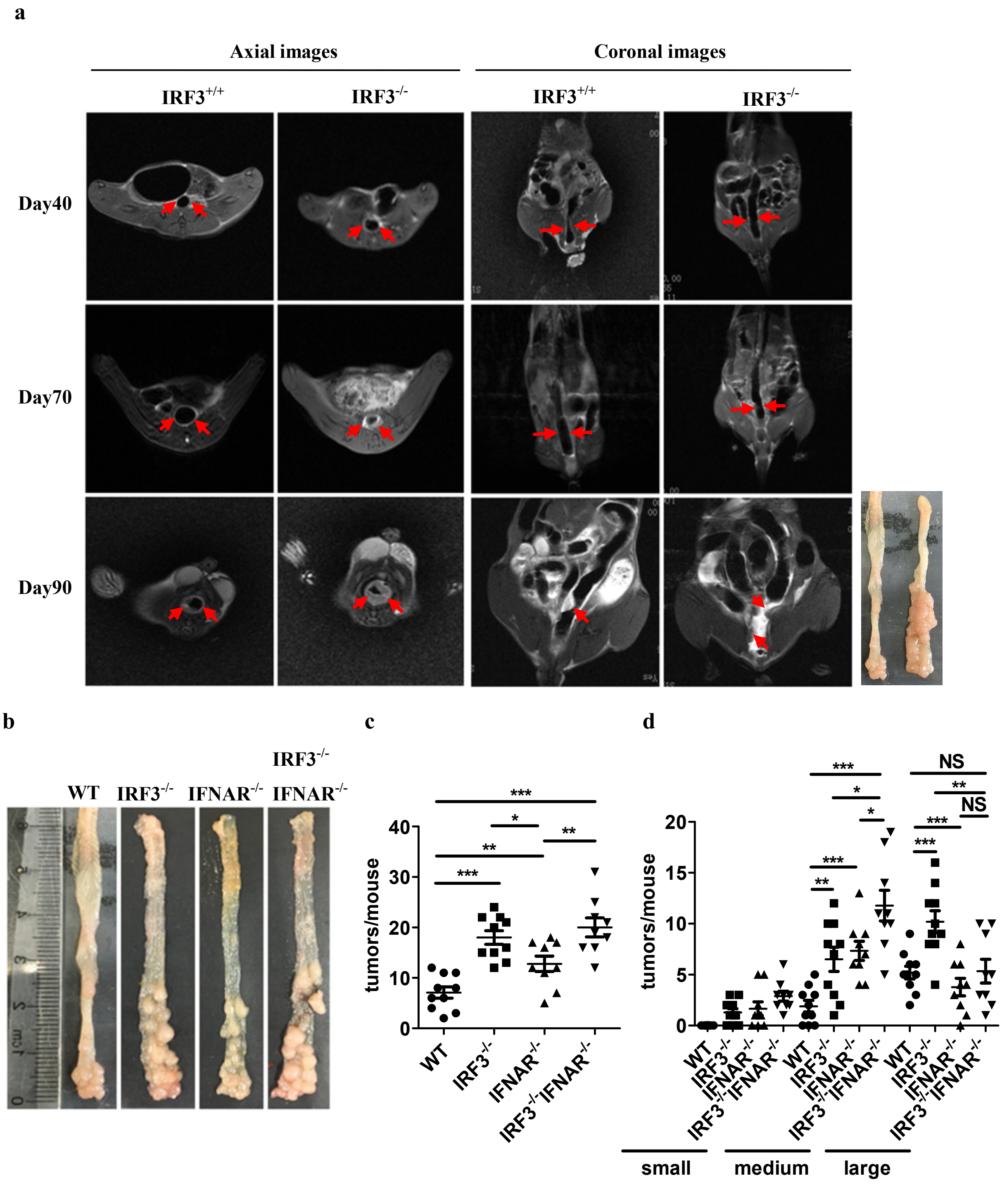
a
Axial images
Coronal images
IRF3+/+
IRF3-/-
IRF3+/+
IRF3-/-
Day40
Day70
Day90
b
WT
IRF3-/-
IFNAR-/-
IRF3-/- IFNAR-/-
c
tumors/mouse
WT
IRF3-/-
IFNAR-/-
IRF3-/-IFNAR-/-
d
tumors/mouse
NS
small
medium
large

e

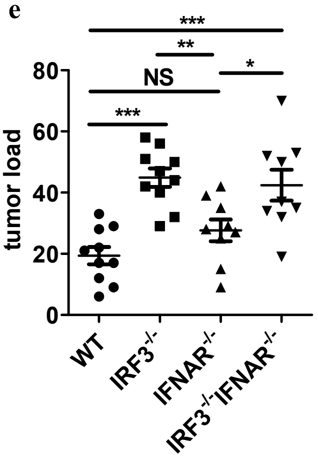


f

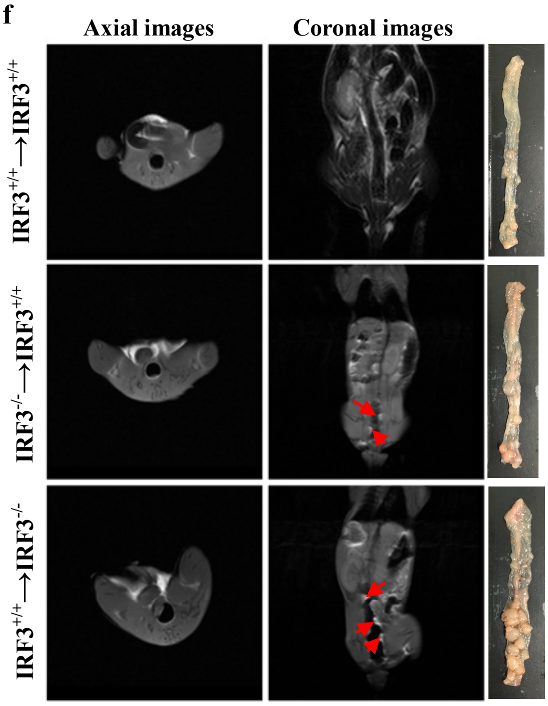


**Supplementary Figure 1. IRF3 prevents colorectal tumorigenesis, which is independent on IFNAR signal pathway. Related to Figure 1 (a)** Representative MRI axial (left) and coronal (right) images on day 40, 70 and 90 after the AOM/DSS treatment. The red arrowhead indicates colon and tumors. **(b)** Representative images of colon tumors from WT, IRF3$^{-/-}$, IFNAR$^{-/-}$ and IRF3$^{-/-}$IFNAR$^{-/-}$ mice on day 90 after AOM/DSS model. **(c-e)** Colon tumors counts, size and tumor load from WT, IRF3$^{-/-}$, IFNAR$^{-/-}$ and IRF3$^{-/-}$IFNAR$^{-/-}$ mice (n=10 mice/group). Representative images of colons at left **(b)** after AOM/DSS model (day 90). **(f)** Representative MRI axial (left) and coronal (right) images of IRF3$^{+/+}$→IRF3$^{+/+}$, IRF3$^{-/-}$→IRF3$^{+/+}$ and IRF3$^{+/+}$→IRF3$^{-/-}$ mice on day 90 after the AOM/DSS treatment. The red arrowhead indicates colon and tumors. Each symbol represents one mouse **(c-e)**. *P< 0.05; **P< 0.01; ***P< 0.001; NS, not statistically significant by two-tailed *t*-test (**c-e**). Data are from two independent experiments (**a-e)** and are presented as mean± s.e.m. in **c-e.**

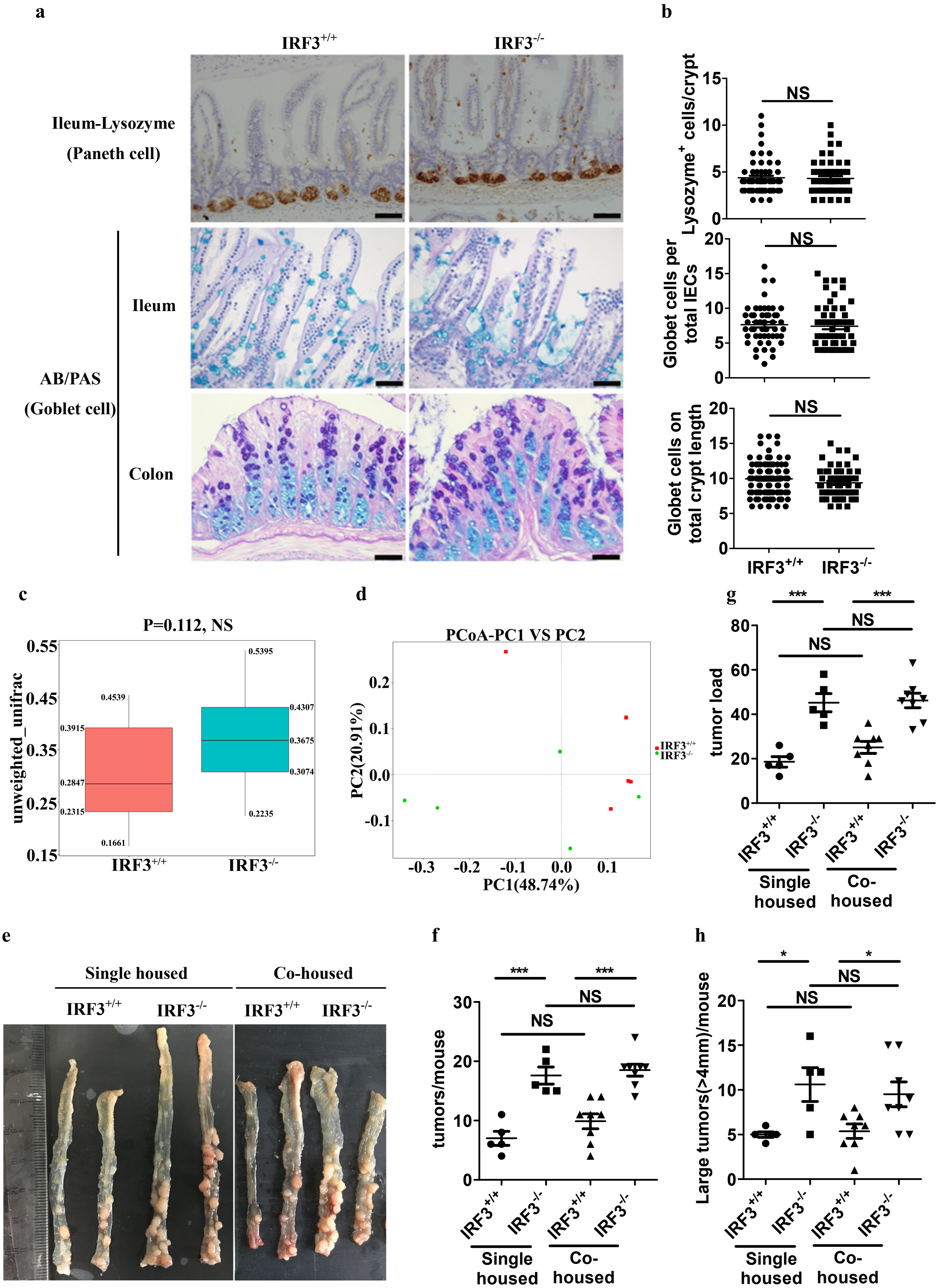

a
IRF3+/+
IRF3-/-
Ileum-Lysozyme
(Paneth cell)
AB/PAS
(Goblet cell)
Ileum
Colon
b
Lysozyme+ cells/crypt
Globet cells per total IECs
Globet cells on total crypt length
NS
IRF3+/+
IRF3-/-
c
P=0.112, NS
unweighted_unifrac
0.5395
0.4539
0.4307
0.3915
0.3675
0.3074
0.2847
0.2315
0.2235
0.1661
d
PCoA-PC1 VS PC2
PC1(48.74%)
PC2(20.91%)
g
tumor load
***
NS
Single housed
Co-housed
e
Single housed
Co-housed
f
tumors/mouse
h
Large tumors(>4mm)/mouse
*

i

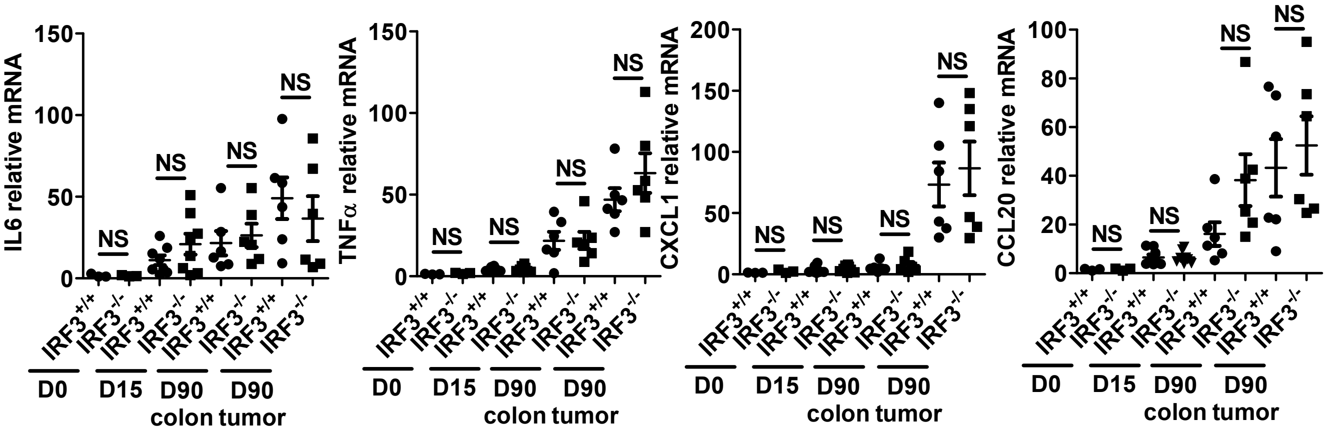
IL6 relative mRNA
TNFα relative mRNA
CXCL1 relative mRNA
CCL20 relative mRNA
NS
IRF3+/+
IRF3-/-
D0
D15
D90
D90
colon tumor

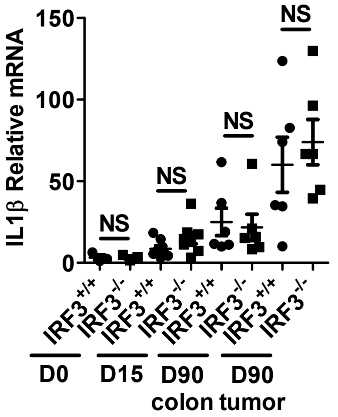
IL1β Relative mRNA
NS
IRF3+/+
IRF3-/-
D0
D15
D90
D90
colon tumor

j

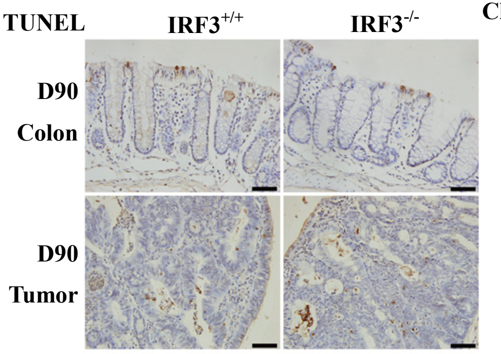
TUNEL
IRF3+/+
IRF3-/-
D90
Colon
D90
Tumor

k

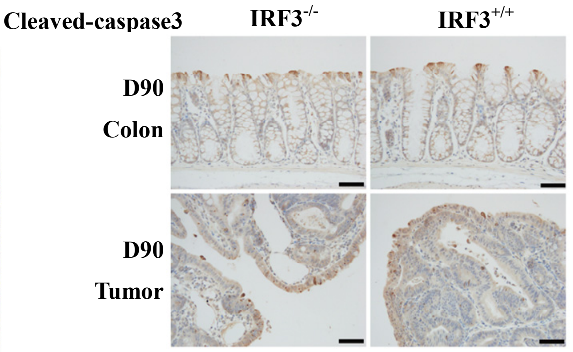
Cleaved-caspase3
IRF3-/-
IRF3+/+
D90
Colon
D90
Tumor

l

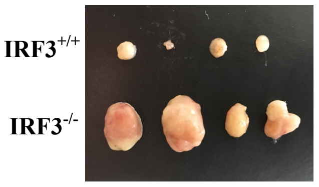
IRF3+/+
IRF3-/-

m

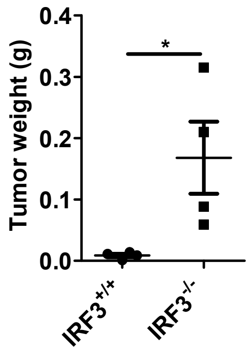
Tumor weight (g)
*
IRF3+/+
IRF3-/-

n

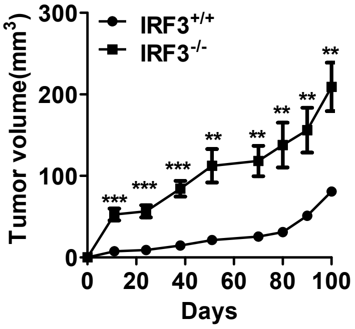
Tumor volume(mm3)
IRF3+/+
IRF3-/-
Days

**Supplementary Figure 2. IRF3 deficiency has no effect on IECs' development, apoptosis, inflammation and gut microbiota. Related to Figure 2. (a-b)** Determination of Paneth cell number per crypt from $IRF3^{+/+}$ and $IRF3^{-/-}$ mice by lysozyme (Lys)-IHC staining. Scoring of goblet cells on Periodic acid Schiff (PAS)-stained sections of ileum and colon (n=3 mice/group, at least 20 crypts in each mouse, and each symbol represents an individual crypt; scale bar, 50 μm). **(c-d)** Analysis of beta diversity between stool samples of separately housed AOM/DSS-treated $IRF3^{+/+}$ (red squares) and $IRF3^{-/-}$ (green squares) mice (day 90, n=4 mice/group) by Box plot and principal coordinates analysis (PCoA) of unweighted UniFrac distances. **(e)** Representative images of colon tumors from separately housed and co-housed $IRF3^{+/+}$ and $IRF3^{-/-}$ mice on day 90 after AOM/DSS model. **(f-h)** The number and size of tumors observed in the colon of separately housed (n=5 mice/group) and co-housed (n=8 mice/group) $IRF3^{+/+}$ and $IRF3^{-/-}$ mice 90 days after injection of AOM. **(i)** Relative expression of genes encoding IL6, TNFα, CXCL1, CCL20 and IL1β in the distal colon and tumor tissue from $IRF3^{+/+}$ and $IRF3^{-/-}$ mice at day 0, 15 and 90 after AOM injection (day 0, n = 3 mice/group; day 15, n =8 mice/group; day 90, n=6 mice/group). **(j)** TUNEL assay of the distal colons and tumors from $IRF3^{+/+}$ and $IRF3^{-/-}$ mice after AOM/DSS model (day 90). **(k)** Cleaved-caspase3 staining of the distal colons and tumors from $IRF3^{+/+}$ and $IRF3^{-/-}$ mice after AOM/DSS model (day 90). **(l)** Images of tumor grafts of $IRF3^{+/+}$ and $IRF3^{-/-}$ tumors in nude mice (n=4 mice/group) at day 100 after implantation. **(m)** Weight of the tumor grafts in **(l). (n)** Volumes of tumor grafts of $IRF3^{+/+}$ and $IRF3^{-/-}$ tumors in nude mice. The absolute tumor volumes are shown on a linear scale on the y axis. Each symbol represents an individual mouse **(c-d, f-i, m).** *P< 0.05; **P< 0.01; ***P< 0.001; NS, not statistically significant by two tailed *t-test* **(b-c, f-i, m).** Data represent two independent experiments and are presented as mean± s.e.m. in **b, f-i, m-n.**

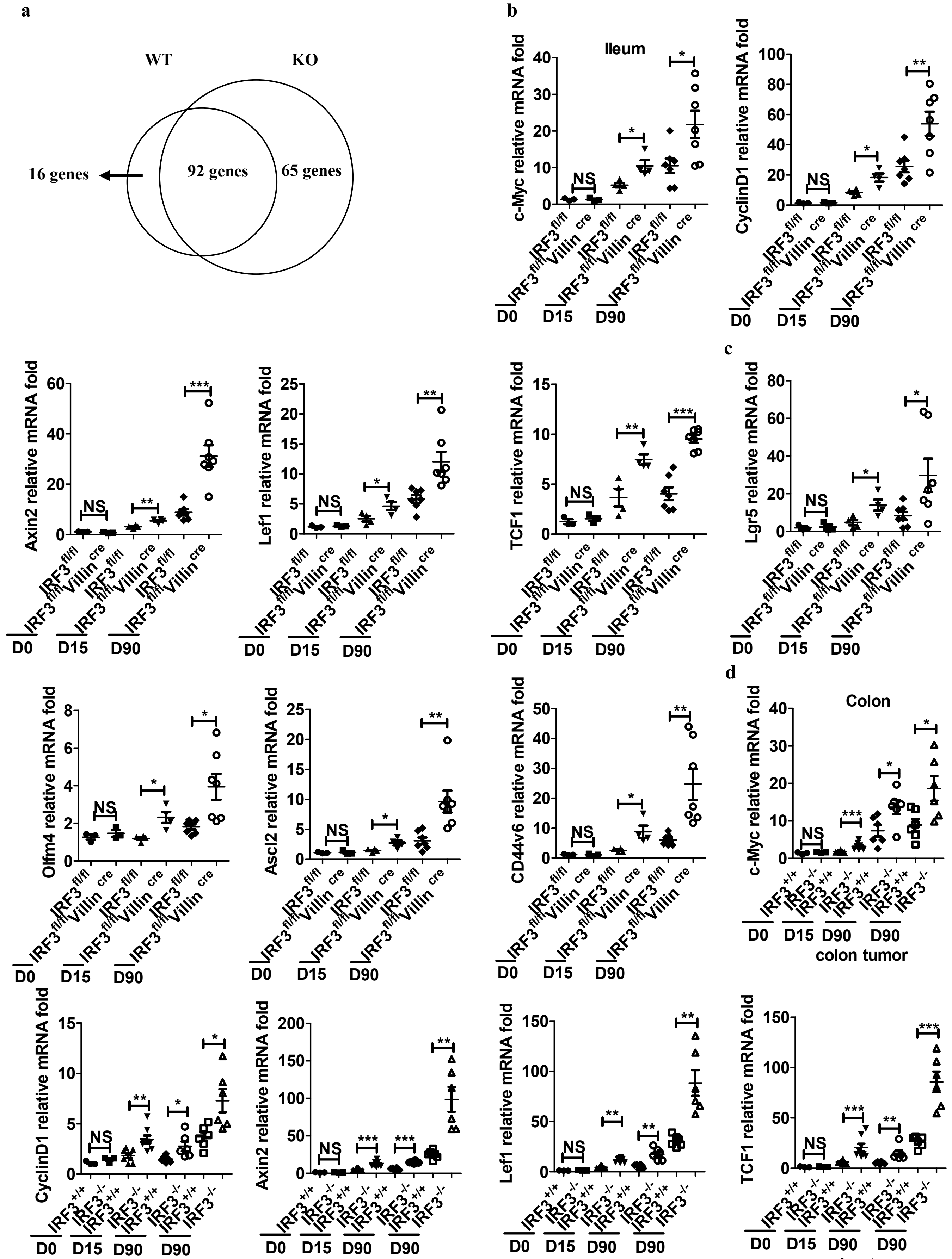
a
WT
KO
16 genes
92 genes
65 genes
b
Ileum
c-Myc relative mRNA fold
CyclinD1 relative mRNA fold
Axin2 relative mRNA fold
Lef1 relative mRNA fold
TCF1 relative mRNA fold
c
Lgr5 relative mRNA fold
Olfm4 relative mRNA fold
Ascl2 relative mRNA fold
CD44v6 relative mRNA fold
IRF3 fl/fl
IRF3 fl/fl Villin cre
D0
D15
D90
NS
d
Colon
c-Myc relative mRNA fold
CyclinD1 relative mRNA fold
Axin2 relative mRNA fold
Lef1 relative mRNA fold
TCF1 relative mRNA fold
IRF3 +/+
IRF3 -/-
D0
D15
D90
D90
colon tumor

e f

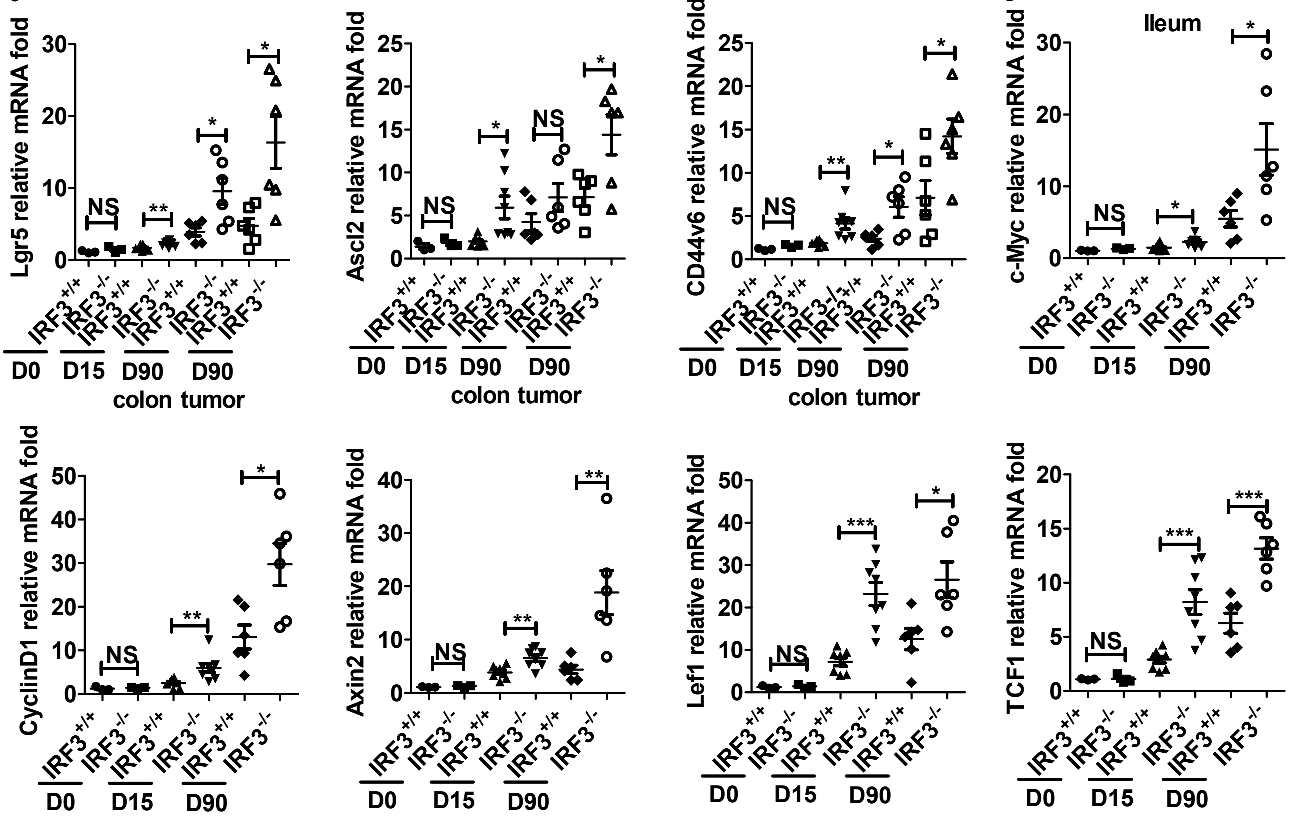

Lgr5 relative mRNA fold
Ascl2 relative mRNA fold
CD44v6 relative mRNA fold
c-Myc relative mRNA fold
Ileum
CyclinD1 relative mRNA fold
Axin2 relative mRNA fold
Lef1 relative mRNA fold
TCF1 relative mRNA fold
NS
*
**
***
IRF3+/+
IRF3-/-
D0
D15
D90
colon tumor


g

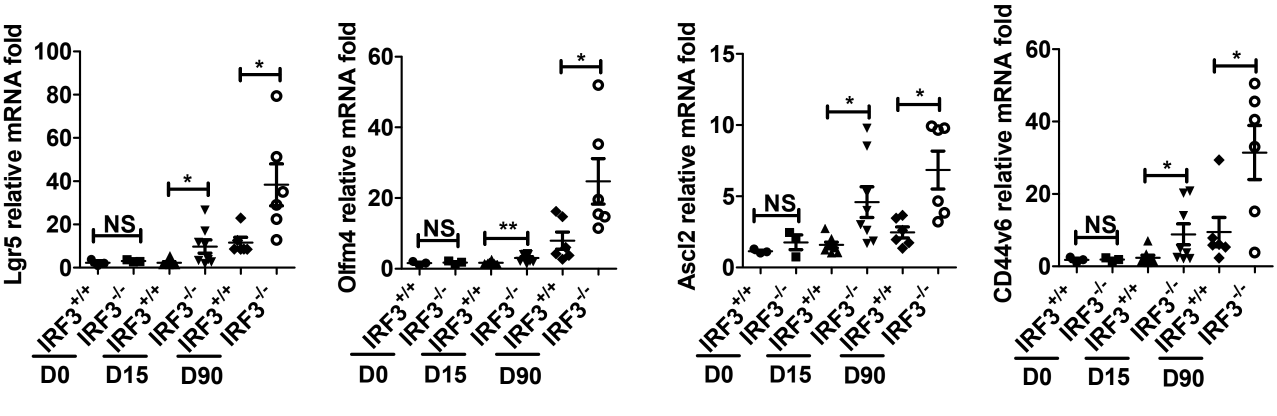

Lgr5 relative mRNA fold
Olfm4 relative mRNA fold
Ascl2 relative mRNA fold
CD44v6 relative mRNA fold
NS
*
**
IRF3+/+
IRF3-/-
D0
D15
D90

h

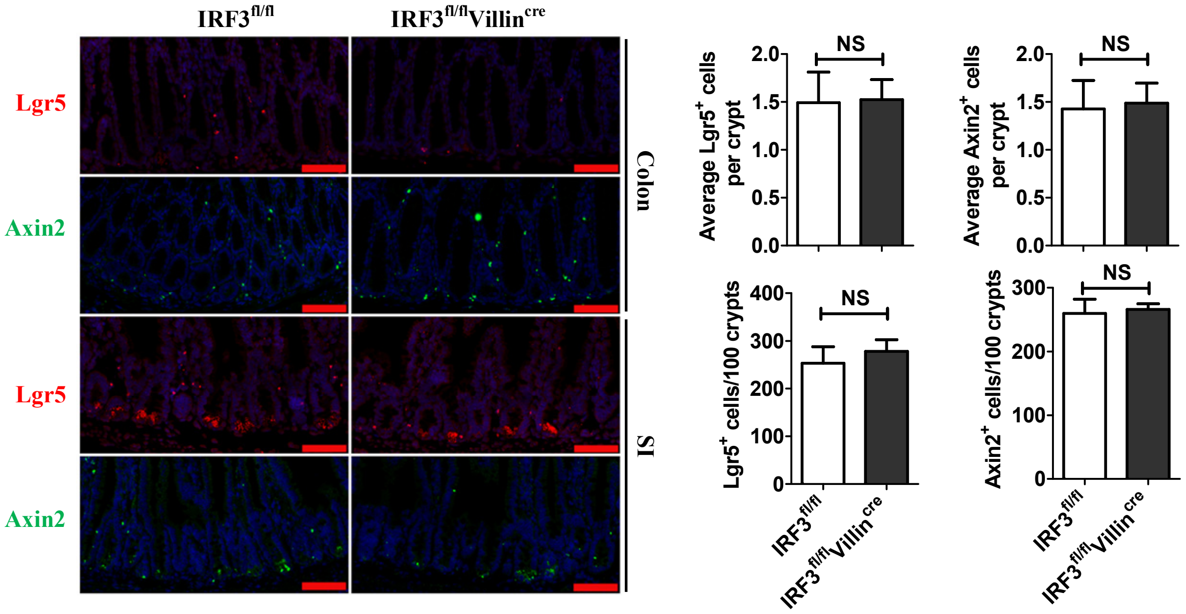

IRF3fl/fl
IRF3fl/flVillincre
Lgr5
Axin2
Lgr5
Axin2
Colon
SI
NS
Average Lgr5+ cells per crypt
2.0
1.5
1.0
0.5
0.0
Average Axin2+ cells per crypt
Lgr5+ cells/100 crypts
400
300
200
100
0
Axin2+ cells/100 crypts
IRF3fl/fl
IRF3fl/flVillin cre


i

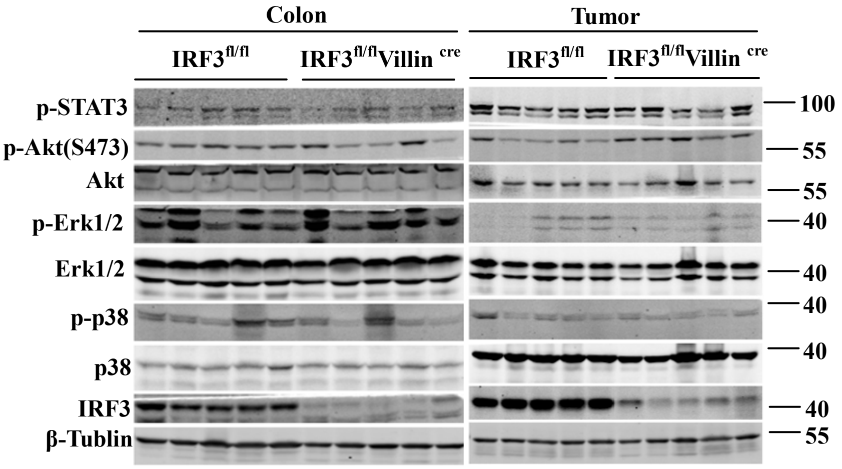

Colon
Tumor
IRF3fl/fl
IRF3fl/flVillin cre
p-STAT3
p-Akt(S473)
Akt
p-Erk1/2
Erk1/2
p-p38
p38
IRF3
β-Tublin
100
55
55
40
40
40
40
40
55


j

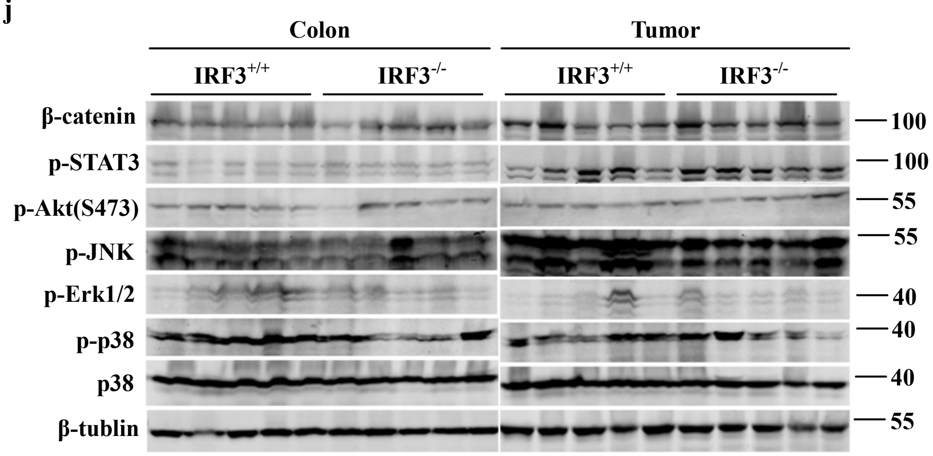

Colon
Tumor
IRF3+/+
IRF3-/-
β-catenin
p-STAT3
p-Akt(S473)
p-JNK
p-Erk1/2
p-p38
p38
β-tublin
100
100
55
55
40
40
40
55

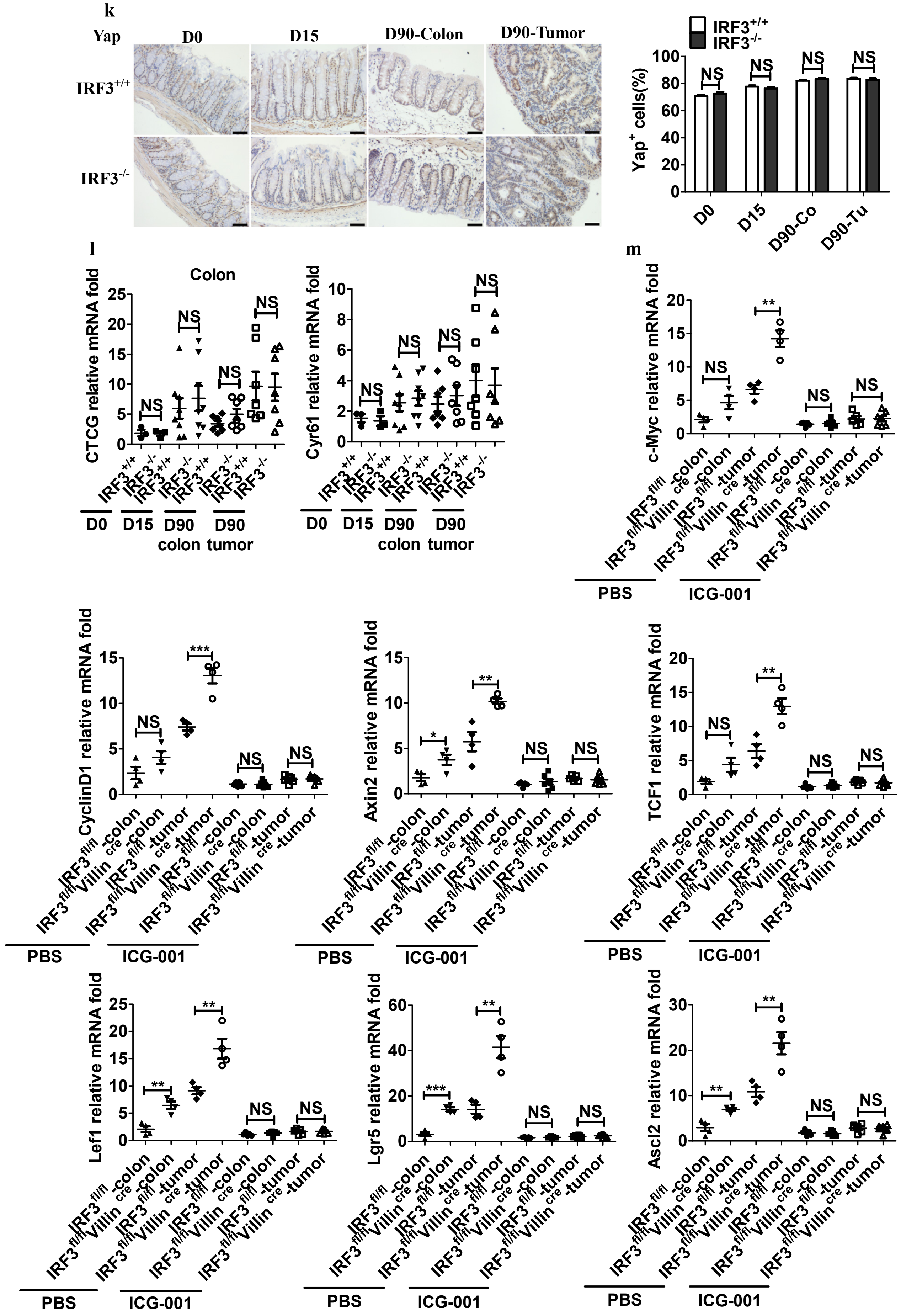
k
Yap
D0
D15
D90-Colon
D90-Tumor
IRF3+/+
IRF3-/-
IRF3+/+
IRF3-/-
Yap+ cells(%)
NS
D0
D15
D90-Co
D90-Tu
l
Colon
CTCG relative mRNA fold
Cyr61 relative mRNA fold
D0
D15
D90 colon
D90 tumor
m
c-Myc relative mRNA fold
CyclinD1 relative mRNA fold
Axin2 relative mRNA fold
TCF1 relative mRNA fold
Lef1 relative mRNA fold
Lgr5 relative mRNA fold
Ascl2 relative mRNA fold
IRF3fl/fl -colon
IRF3fl/fl Villin cre -colon
IRF3fl/fl -tumor
IRF3fl/fl Villin cre -tumor
PBS
ICG-001

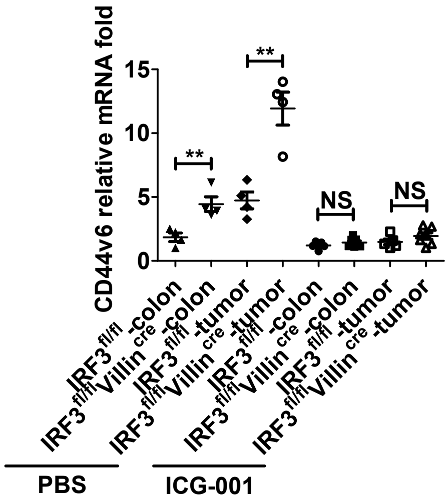
CD44v6 relative mRNA fold
15
10
5
0
**
**
NS
NS
IRF3 fl/fl -colon
IRF3 fl/fl Villin cre -colon
IRF3 fl/fl -tumor
IRF3 fl/fl Villin cre -tumor
IRF3 fl/fl -colon
IRF3 fl/fl Villin cre -colon
IRF3 fl/fl -tumor
IRF3 fl/fl Villin cre -tumor
PBS
ICG-001

n

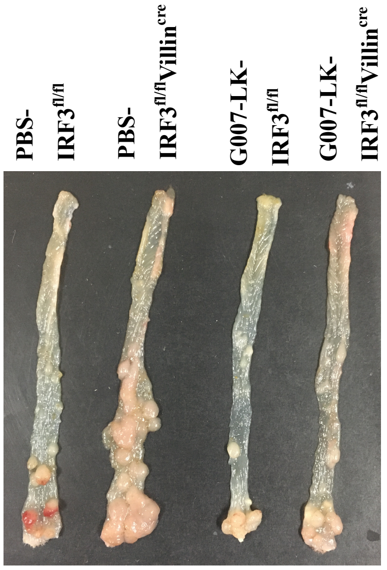
PBS-
IRF3 fl/fl
PBS-
IRF3 fl/fl Villin cre
G007-LK-
IRF3 fl/fl
G007-LK-
IRF3 fl/fl Villin cre

o

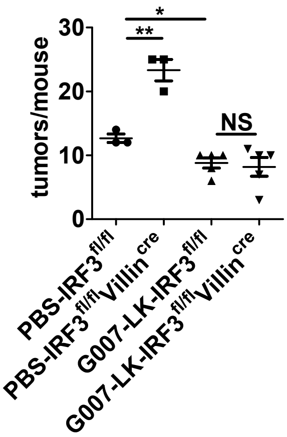
tumors/mouse
30
20
10
0
*
**
NS
PBS-IRF3 fl/fl
PBS-IRF3 fl/fl Villin cre
G007-LK-IRF3 fl/fl
G007-LK-IRF3 fl/fl Villin cre

p

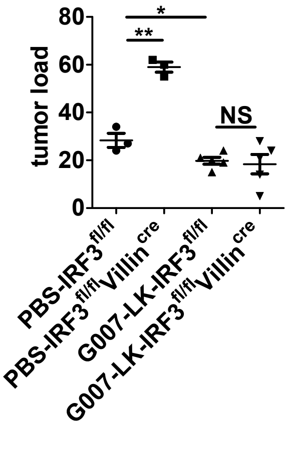
tumor load
80
60
40
20
0
*
**
NS
PBS-IRF3 fl/fl
PBS-IRF3 fl/fl Villin cre
G007-LK-IRF3 fl/fl
G007-LK-IRF3 fl/fl Villin cre

q

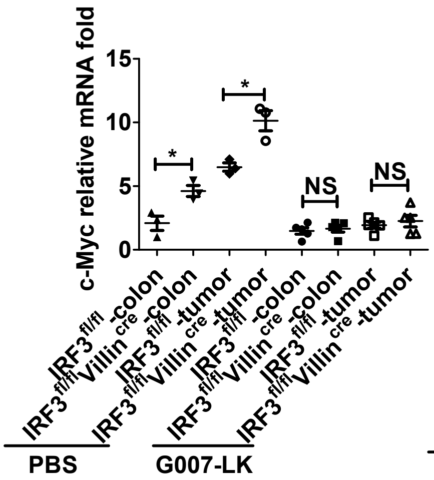
c-Myc relative mRNA fold
15
10
5
0
*
*
NS
NS
IRF3 fl/fl -colon
IRF3 fl/fl Villin cre -colon
IRF3 fl/fl -tumor
IRF3 fl/fl Villin cre -tumor
IRF3 fl/fl -colon
IRF3 fl/fl Villin cre -colon
IRF3 fl/fl -tumor
IRF3 fl/fl Villin cre -tumor
PBS
G007-LK

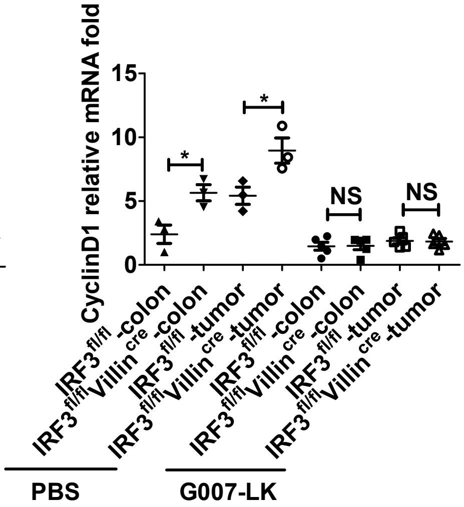
CyclinD1 relative mRNA fold
15
10
5
0
*
*
NS
NS
IRF3 fl/fl -colon
IRF3 fl/fl Villin cre -colon
IRF3 fl/fl -tumor
IRF3 fl/fl Villin cre -tumor
IRF3 fl/fl -colon
IRF3 fl/fl Villin cre -colon
IRF3 fl/fl -tumor
IRF3 fl/fl Villin cre -tumor
PBS
G007-LK

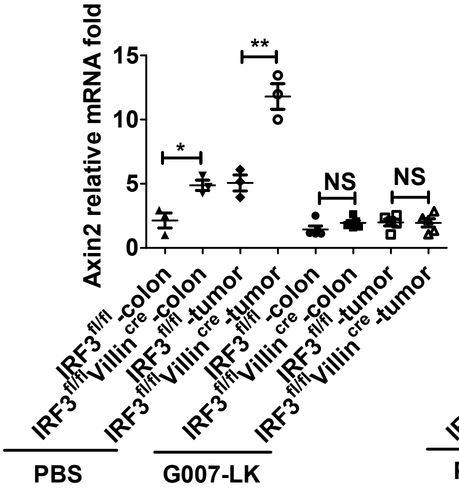
Axin2 relative mRNA fold
15
10
5
0
*
**
NS
NS
IRF3 fl/fl -colon
IRF3 fl/fl Villin cre -colon
IRF3 fl/fl -tumor
IRF3 fl/fl Villin cre -tumor
IRF3 fl/fl -colon
IRF3 fl/fl Villin cre -colon
IRF3 fl/fl -tumor
IRF3 fl/fl Villin cre -tumor
PBS
G007-LK

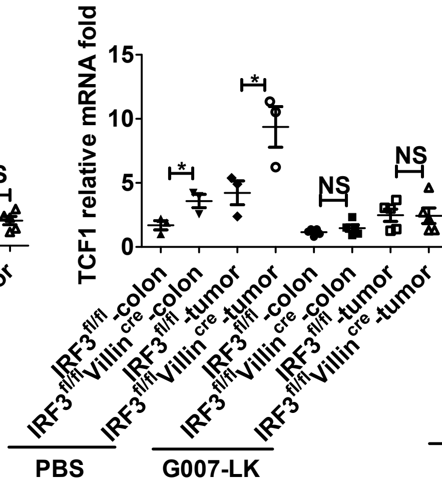
TCF1 relative mRNA fold
15
10
5
0
*
*
NS
NS
IRF3 fl/fl -colon
IRF3 fl/fl Villin cre -colon
IRF3 fl/fl -tumor
IRF3 fl/fl Villin cre -tumor
IRF3 fl/fl -colon
IRF3 fl/fl Villin cre -colon
IRF3 fl/fl -tumor
IRF3 fl/fl Villin cre -tumor
PBS
G007-LK

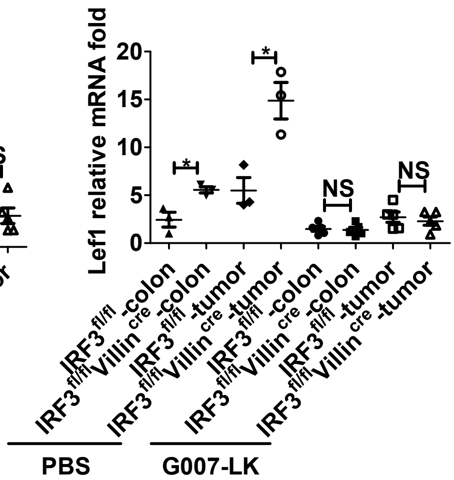
Lef1 relative mRNA fold
20
15
10
5
0
*
*
NS
NS
IRF3 fl/fl -colon
IRF3 fl/fl Villin cre -colon
IRF3 fl/fl -tumor
IRF3 fl/fl Villin cre -tumor
IRF3 fl/fl -colon
IRF3 fl/fl Villin cre -colon
IRF3 fl/fl -tumor
IRF3 fl/fl Villin cre -tumor
PBS
G007-LK

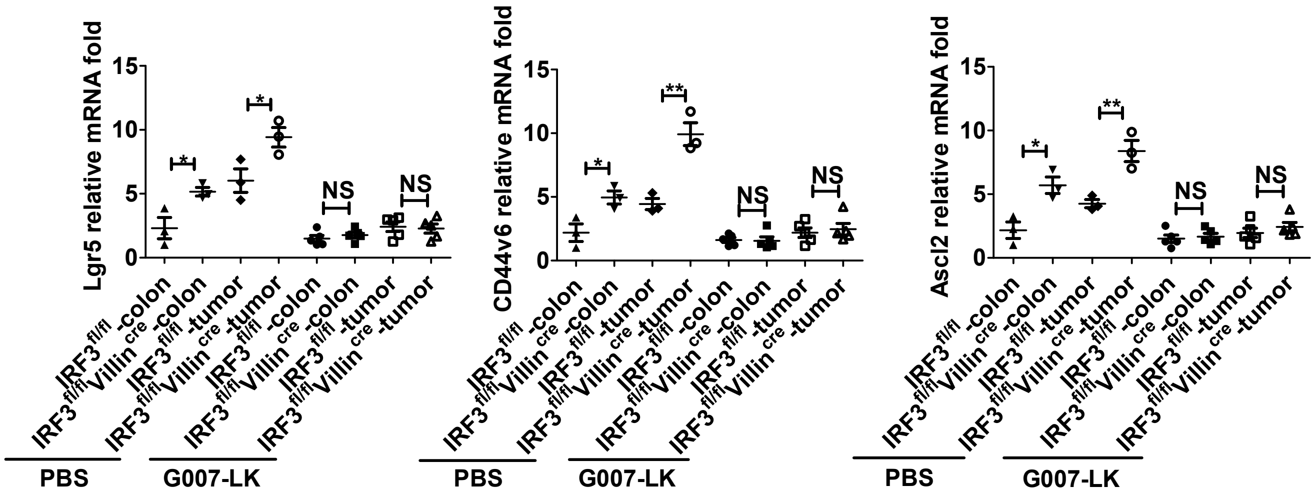
Lgr5 relative mRNA fold
CD44v6 relative mRNA fold
Ascl2 relative mRNA fold
15
10
5
0
*
**
NS
IRF3 fl/fl -colon
IRF3 fl/fl Villin cre -colon
IRF3 fl/fl -tumor
IRF3 fl/fl Villin cre -tumor
PBS
G007-LK

**Supplementary Figure 3. IRF3 deficiency specifically affects Wnt signaling pathway in the colon tissue. Related to Figure 3. (a)** Venn diagram representation of the genes analyzed from the RNA-seq results: 16 genes that up-regulated in tumor tissue only in "WT", 92 genes that up-regulated both in "WT" and "KO" and 65 genes that up-regulated only in "KO". **(b-c)** Real time qPCR analysis for expression of the Wnt target and associated genes in ileum from $IRF3^{fl/fl}$ and $IRF3^{fl/fl}Villin^{cre}$ upon AOM/DSS treatment (day 0, n = 3 mice/group; day 15, n =4 mice/group; day 90, n =7 mice/group) mice. **(d-e)** Real time qPCR analysis for expression of the Wnt target and associated genes in the distal colon and tumors from $IRF3^{+/+}$ and $IRF3^{-/-}$ mice upon AOM/DSS treatment (day 0, n = 3 mice/group; day 15, n =8 mice/group; day 90, n=6 mice/group) mice. **(f-g)** Real time qPCR analysis for expression of the Wnt target and associated genes in ileum from $IRF3^{+/+}$ and $IRF3^{-/-}$ (day 0, n = 3 mice/group; day 15, n =8 mice/group; day 90, n =6 mice/group) mice upon AOM/DSS treatment. **(h)** Standardized Lgr5 and Axin2 ISH of colon and the small intestine (SI) tissues of $IRF3^{fl/fl}$ and $IRF3^{fl/fl}Villin^{cre}$ mice (the left). And quantitative analysis of Lgr5 and Axin2 RNA level of colon and the small intestine (SI) tissues of $IRF3^{fl/fl}$ and $IRF3^{fl/fl}Villin^{cre}$ mice (the right, n=3 mice each group). Red scale bar, 50 μm. **(i)** Immunoblot analysis of p-STAT3, p-Akt, p-Erk1/2 and p-p38 in the distal colon and tumor tissues from $IRF3^{fl/fl}$ and $IRF3^{fl/fl}Villin^{cre}$ mice (n=5 mice/group) on day 90 after AOM/DSS treatment. **(j)** Immunoblot analysis of β-catenin p-STAT3, p-Akt, p-JNK, p-Erk1/2 and p-p38 in the distal colon and tumor tissues from $IRF3^{+/+}$ and $IRF3^{-/-}$ mice (n=5 mice/group) on day 90 after AOM/DSS treatment. **(k)** Immunohistochemical analysis of Yap in the colon and tumors from $IRF3^{+/+}$ and $IRF3^{-/-}$ mice after treatment with AOM/DSS (day 0, 15 and 90, n=3 mice/group，at least 20 crypts in each mouse, and each symbol represents an individual crypt). **(l)** Real time qPCR analysis for the Yap target genes expression in the distal colon and tumor from $IRF3^{+/+}$ and $IRF3^{-/-}$ mice upon AOM/DSS treatment (day 0, n = 3 mice/group; day15, n=8 mice/group; day 90, n=7 mice/group). **(m)** Real time qPCR analysis for the Wnt target and associated genes expression in the distal colon and tumors from AOM/DSS-treated mice with PBS (n=4 mice/group) or ICG-001 ($IRF3^{fl/fl}$ mice, n=5; $IRF3^{fl/fl}Villin^{cre}$ mice, n=7) treatment (300 mg/kg per day, orally, once daily, six times one week until day 90). **(n-p)** Colon tumors counts **(o)** and tumor load **(p)** in AOM/DSS-treated mice with PBS or G007-LK treatment (50 mg/kg per day, once every three days for the last 10 weeks of the AOM/DSS model), representative images of colons at **(n)** (PBS group, n = 3 mice/group; G007-LK group, n = 5 mice/group). **(q)** Real time qPCR analysis for the Wnt target and associated genes expression in the distal colon and tumors from AOM/DSS-treated mice with PBS (n=3 mice/group) or G007-LK (n = 5 mice/group) treatment (50 mg/kg per day, orally, once every three days, for the last 10 weeks of the AOM/DSS model). Each symbol represents an individual mouse **(b-i, m-n)**. **P< 0.01; ***P< 0.001; NS, not statistically significant by two tailed t--test **(b-h, l-m, o-q)**. Data represent two independent experiments **(c-q)** and are presented as mean± s.e.m. in **b-h, l-m, o-q**.

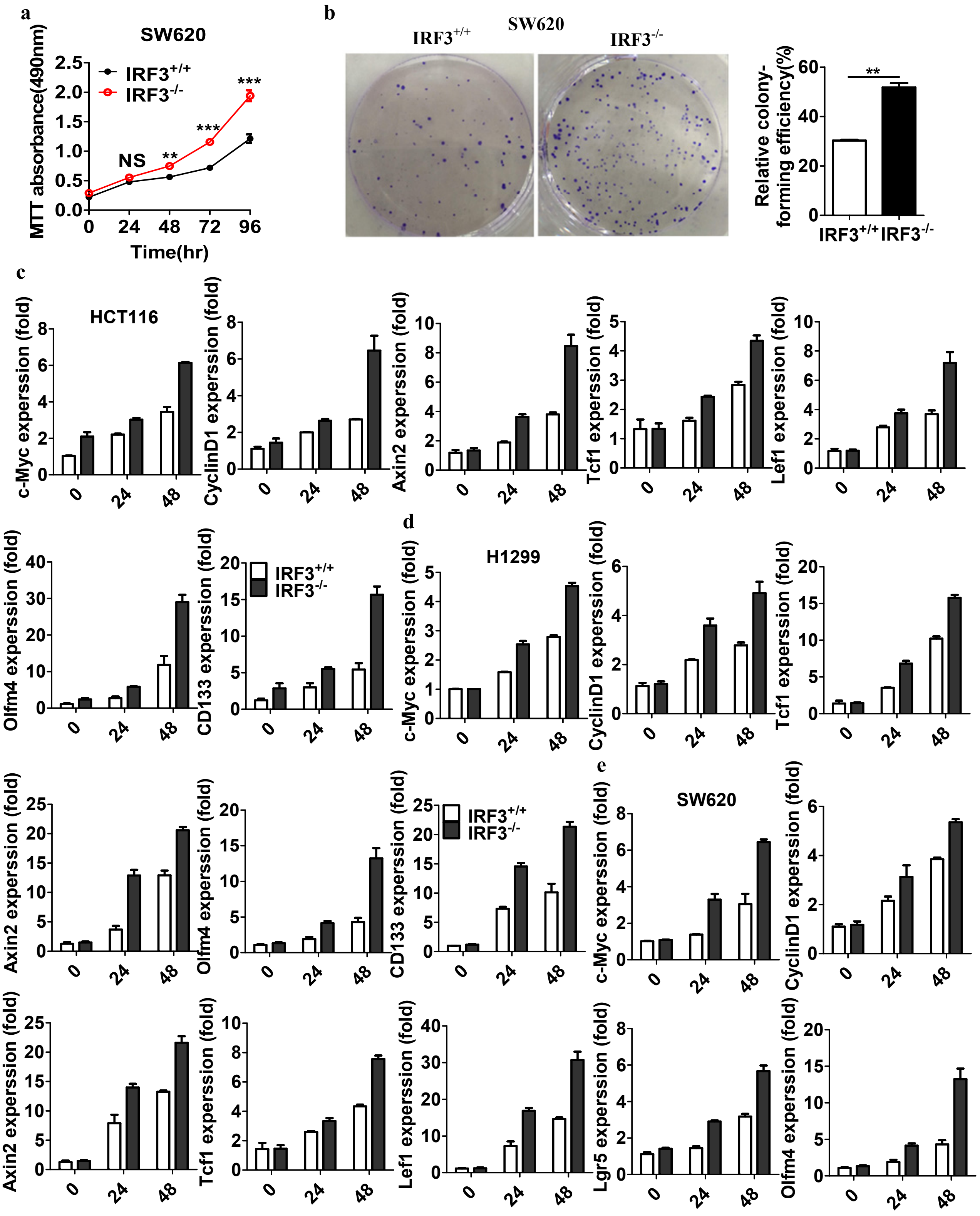
a
SW620
IRF3+/+
IRF3-/-
MTT absorbance(490nm)
Time(hr)
NS
**
***
***
b
SW620
IRF3+/+
IRF3-/-
Relative colony-forming efficiency(%)
**
c
HCT116
c-Myc expersssion (fold)
CyclinD1 expression (fold)
Axin2 expersssion (fold)
Tcf1 expersssion (fold)
Lef1 expersssion (fold)
Olfm4 expersssion (fold)
CD133 expersssion (fold)
IRF3+/+
IRF3-/-
d
H1299
c-Myc expersssion (fold)
CyclinD1 expersssion (fold)
Tcf1 expersssion (fold)
Axin2 expersssion (fold)
Olfm4 expersssion (fold)
CD133 expersssion (fold)
IRF3+/+
IRF3-/-
e
SW620
c-Myc expersssion (fold)
CyclinD1 expersssion (fold)
Axin2 expersssion (fold)
Tcf1 expersssion (fold)
Lef1 expersssion (fold)
Lgr5 expersssion (fold)
Olfm4 expersssion (fold)

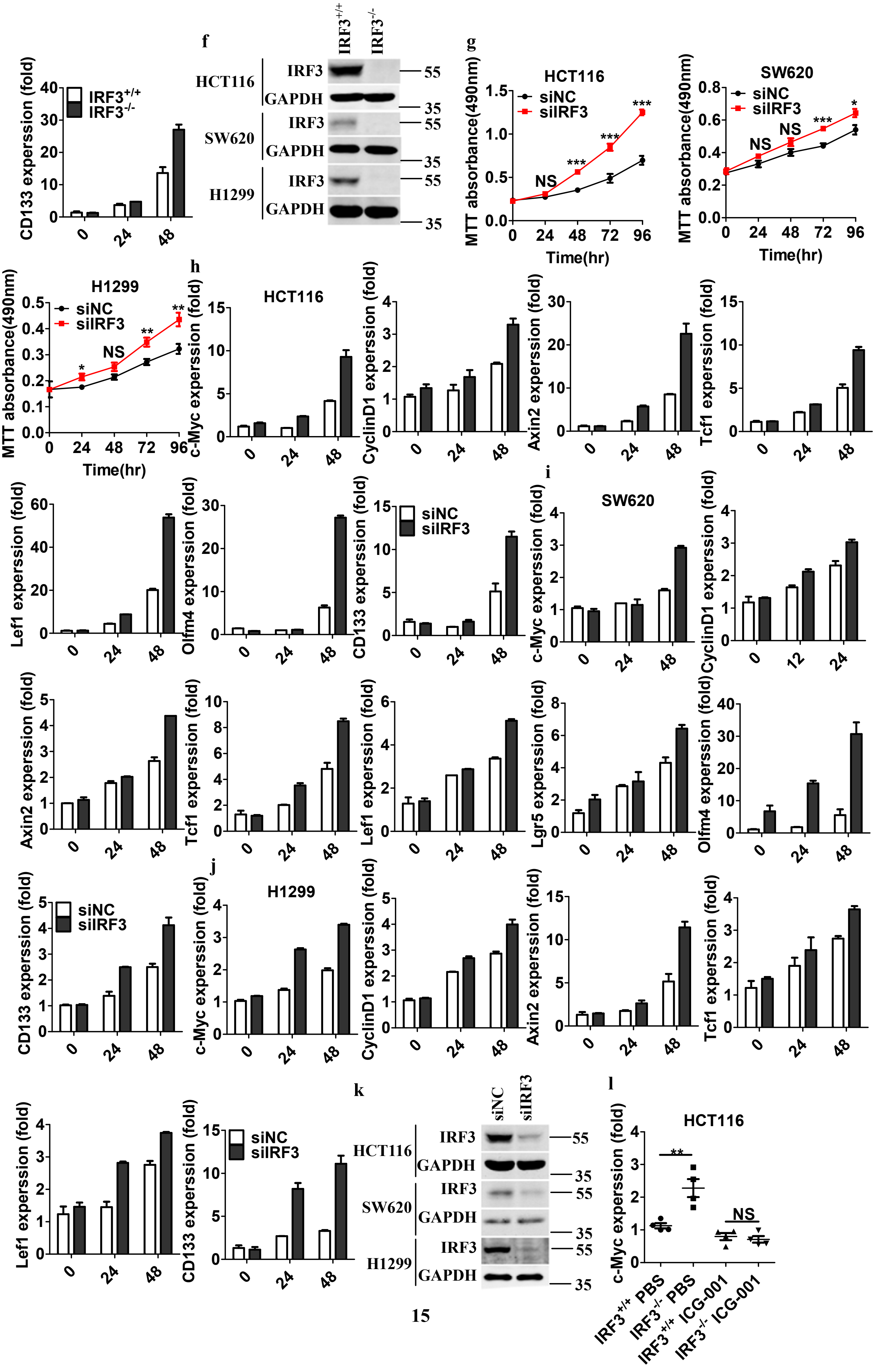
f
IRF3+/+
IRF3-/-
HCT116
SW620
H1299
IRF3
GAPDH
55
35
g
HCT116
SW620
H1299
siNC
siIRF3
MTT absorbance(490nm)
Time(hr)
NS
h
HCT116
CD133 expression (fold)
c-Myc expression (fold)
CyclinD1 expression (fold)
Axin2 expression (fold)
Tcf1 expression (fold)
Lef1 expression (fold)
Olfm4 expression (fold)
Lgr5 expression (fold)
i
SW620
j
H1299
k
l
HCT116
IRF3+/+ PBS
IRF3-/- PBS
IRF3+/+ ICG-001
IRF3-/- ICG-001
**
NS

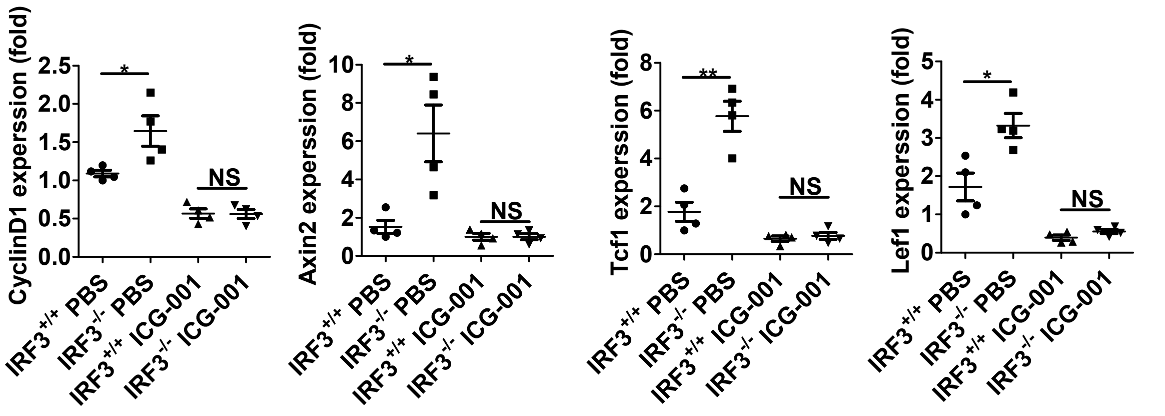
CyclinD1 expression (fold)
Axin2 expression (fold)
Tcf1 expression (fold)
Lef1 expression (fold)
Olfm4 expression (fold)
CD133 expression (fold)
IRF3+/+ PBS
IRF3-/- PBS
IRF3+/+ ICG-001
IRF3-/- ICG-001
*
**
NS

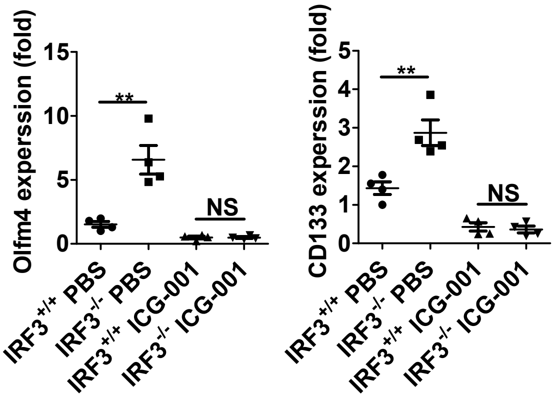

m

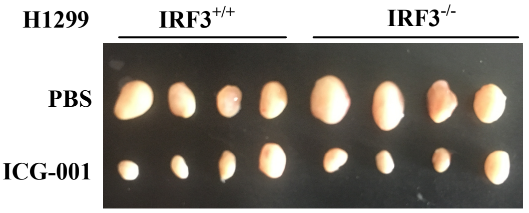
H1299
IRF3+/+
IRF3-/-
PBS
ICG-001

n

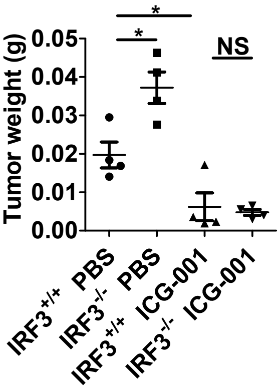
Tumor weight (g)
IRF3+/+ PBS
IRF3-/- PBS
IRF3+/+ ICG-001
IRF3-/- ICG-001
*
NS

o

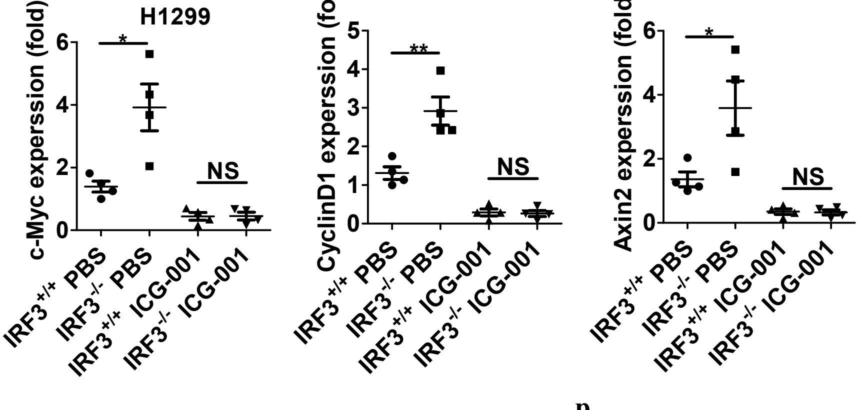
H1299
c-Myc expression (fold)
CyclinD1 expression (fold)
Axin2 expression (fold)
Tcf1 experssion (fold)
Lef1 experssion (fold)
CD133 experssion (fold)
IRF3+/+ PBS
IRF3-/- PBS
IRF3+/+ ICG-001
IRF3-/- ICG-001
*
**
NS

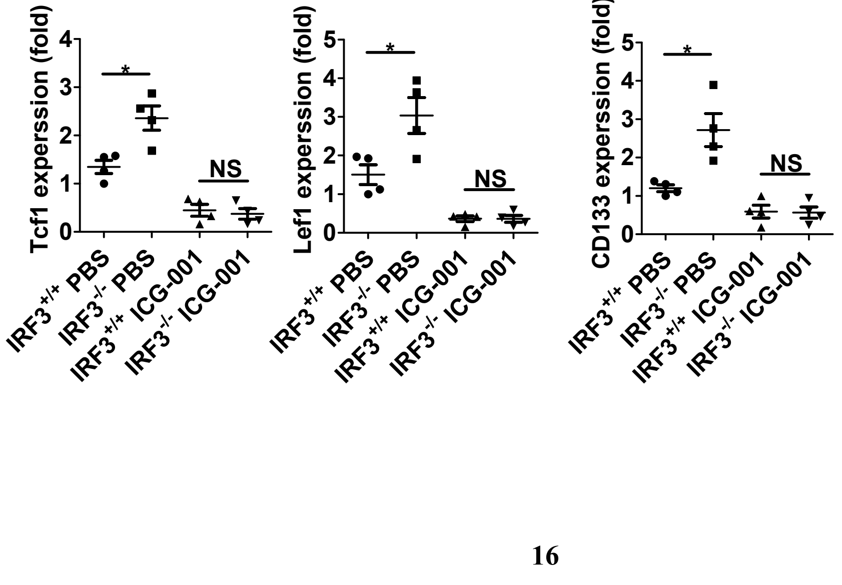

p

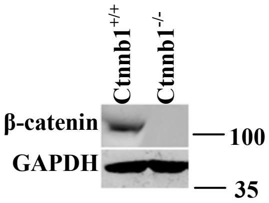
Ctnnb1+/+
Ctnnb1-/-
β-catenin
GAPDH
100
35

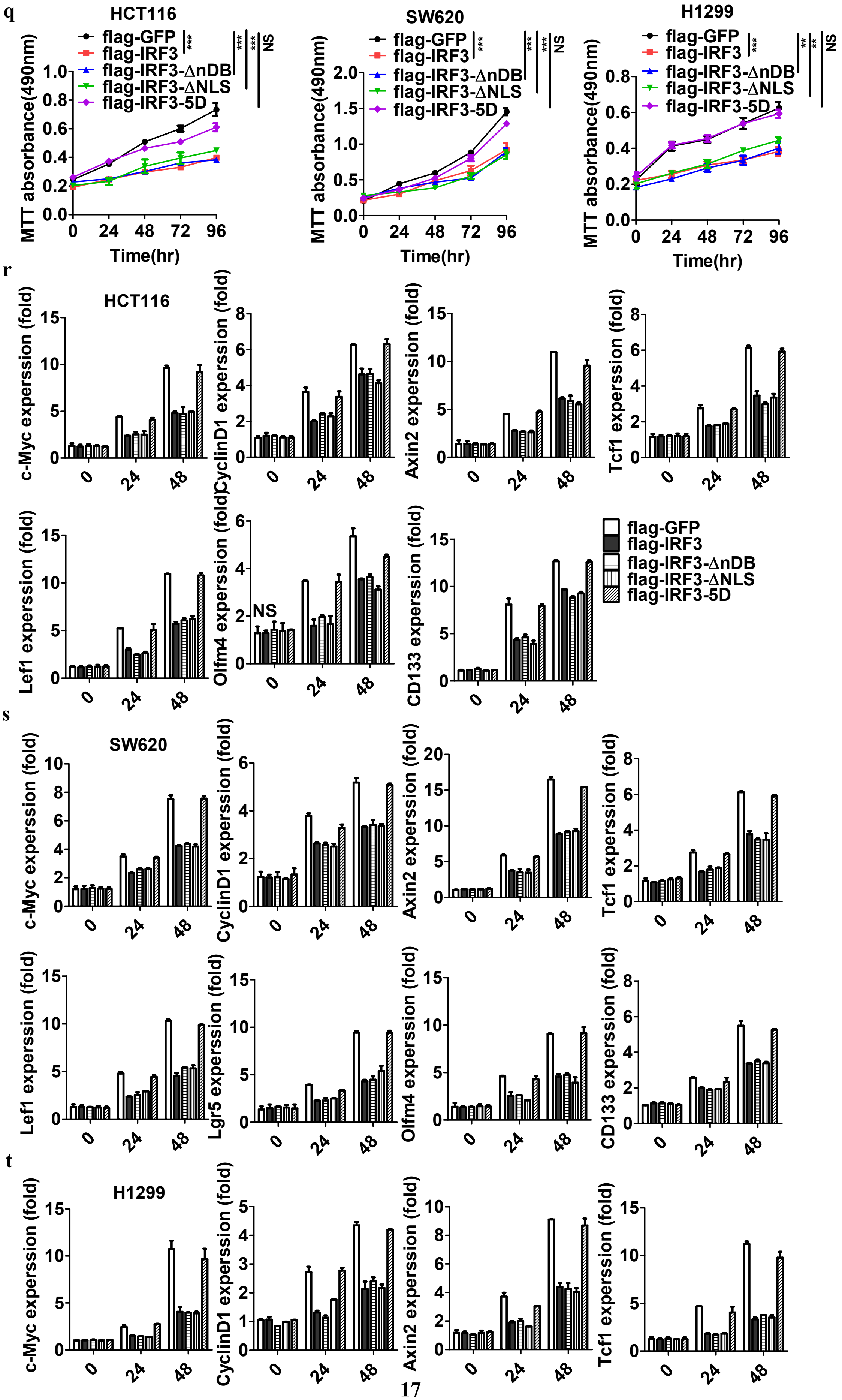
q
HCT116
SW620
H1299
flag-GFP
flag-IRF3
flag-IRF3-ΔnDB
flag-IRF3-ΔNLS
flag-IRF3-5D
***
**
NS
MTT absorbance(490nm)
Time(hr)
r
HCT116
c-Myc expression (fold)
CyclinD1 experssion (fold)
Axin2 expression (fold)
Tcf1 expression (fold)
Lef1 expression (fold)
Olfm4 expression (fold)
CD133 expression (fold)
NS
s
SW620
c-Myc experssion (fold)
CyclinD1 experssion (fold)
Axin2 expression (fold)
Tcf1 expression (fold)
Lef1 expression (fold)
Lgr5 expression (fold)
Olfm4 expression (fold)
CD133 expression (fold)
t
H1299
c-Myc expression (fold)
CyclinD1 expression (fold)
Axin2 experssion (fold)
Tcf1 expression (fold)

u

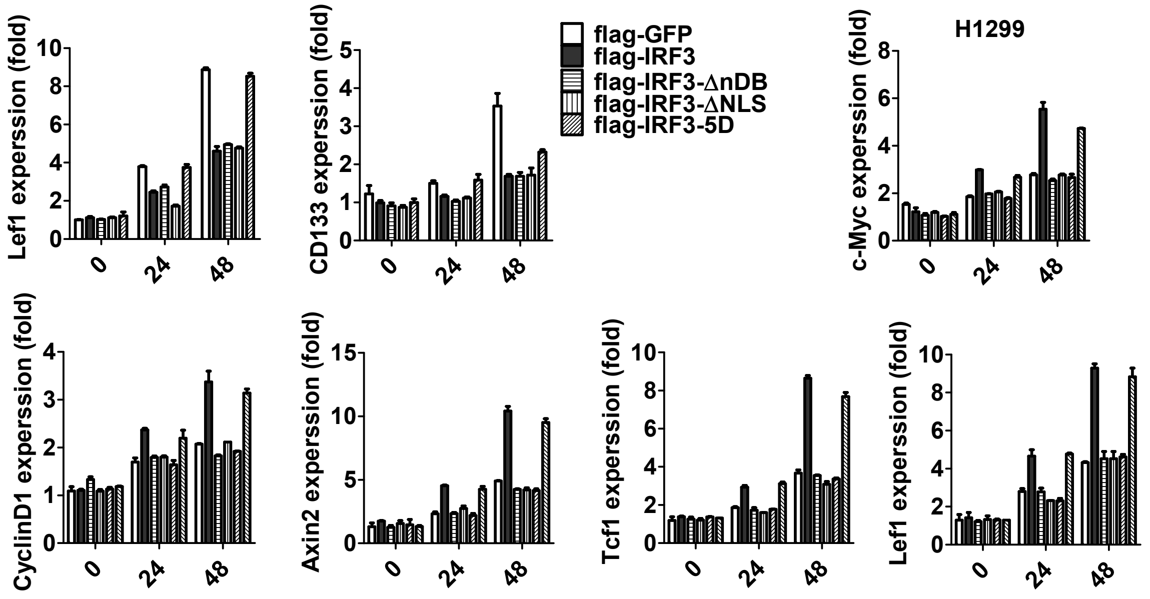

H1299
flag-GFP
flag-IRF3
flag-IRF3-ΔnDB
flag-IRF3-ΔNLS
flag-IRF3-5D
Lef1 expression (fold)
CD133 expression (fold)
c-Myc expression (fold)
CyclinD1 expression (fold)
Axin2 expression (fold)
Tcf1 expression (fold)
Lef1 expression (fold)
0
24
48


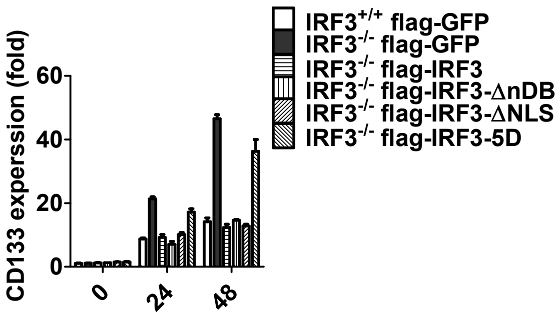

IRF3+/+ flag-GFP
IRF3-/- flag-GFP
IRF3-/- flag-IRF3
IRF3-/- flag-IRF3-ΔnDB
IRF3-/- flag-IRF3-ΔNLS
IRF3-/- flag-IRF3-5D
CD133 expression (fold)
0
24
48


v

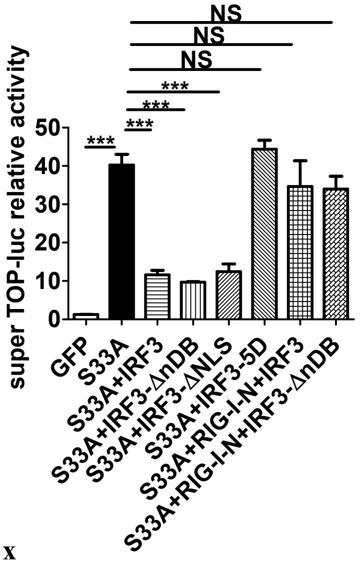

super TOP-luc relative activity
NS
NS
NS
***
***
***
***
GFP
S33A
S33A+IRF3
S33A+IRF3-ΔnDB
S33A+IRF3-ΔNLS
S33A+IRF3-5D
S33A+RIG-I-N+IRF3
S33A+RIG-I-N+IRF3-ΔnDB


w

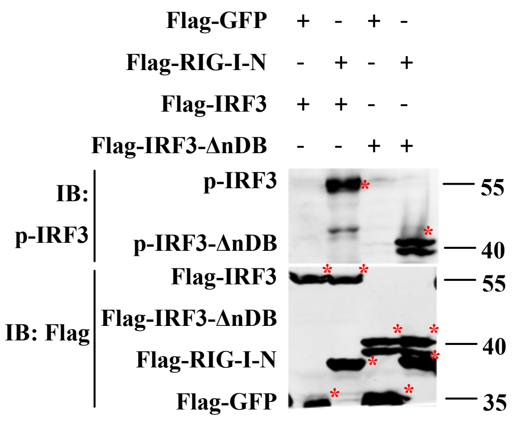

Flag-GFP + - + -
Flag-RIG-I-N - + - +
Flag-IRF3 + + - -
Flag-IRF3-ΔnDB - - + +
IB: p-IRF3
p-IRF3
p-IRF3-ΔnDB
IB: Flag
Flag-IRF3
Flag-IRF3-ΔnDB
Flag-RIG-I-N
Flag-GFP
55
40
55
40
35


x

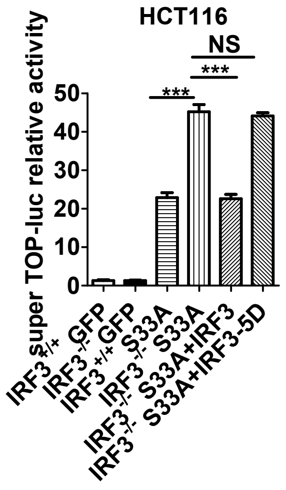

HCT116
super TOP-luc relative activity
NS
***
***
IRF3+/+ GFP
IRF3-/- GFP
IRF3+/+ S33A
IRF3-/- S33A
IRF3-/- S33A+IRF3
IRF3-/- S33A+IRF3-5D

**Supplementary Figure 4. The cytoplasmic IRF3 in resting state inhibits the cell proliferation and Wnt/β-catenin pathway in HCT116, SW620 and H1299 cell lines. Related to Figure 4. (a) P**roliferation of the IRF3$^{+/+}$ and IRF3$^{-/-}$SW620 cells. **(b)** Colony-formation experiment of the IRF3$^{+/+}$ and IRF3$^{-/-}$ SW620 cells. **(c-e)** Real time qPCR analysis for the Wnt target and associated genes in IRF3$^{+/+}$ and IRF3$^{-/-}$ HCT116 **(c)**, H1299 **(d)**, and SW620 **(e)** cells treated as in cell proliferation assay (described in the Materials and Methods). **(f)** Immunoblot analysis confirmed the deficiency of IRF3 in HCT116, SW620 and H1299 cells. **(g)** Proliferation of the HCT116, SW620, and H1299 cells treated with IRF3-specific (siIRF3) or scrambled (siNC) short interfering RNAs. **(h-j)** Real time qPCR analysis for the Wnt target and associated genes in HCT116 **(h)**, SW620 **(i)**, and H1299 **(j)** cells treated with siIRF3 or siNC. **(k)** Immunoblot analysis of IRF3 in siNC or siIRF3 transfected HCT116, SW620, and H1299 cells. **(l)** Real time qPCR analysis for the Wnt target and associated genes in tumors formed by IRF3$^{+/+}$ and IRF3$^{-/-}$ HCT116 cells with or without ICG-001 treatment. **(m-n)** Representative images of tumors from subcutaneous tumor formation assay in nude mice **(m)**. Subcutaneous tumor formation assay in nude mice was with $4\times10^6$ IRF3$^{+/+}$ or IRF3$^{-/-}$ H1299 cells per mouse. After one week of the injection, PBS or ICG-001 treatment (200 mg/kg, i.v., once daily) was applied in mice until the end of the model. Tumor weight for each group (n=4) was plotted **(n)**. **(o)** Real time qPCR analysis for the Wnt target and associated genes in tumors from **(m)**. **(p)** Immunoblot analysis confirmed the deficiency of β-catenin in HCT116 cells. **(q)** Proliferation of HCT116, SW620, and H1299 cells transfected with the indicated plasmids expressing backbone, IRF3 or IRF3 mutants. **(r-t)** Real time qPCR analysis for the Wnt target and associated genes in HCT116 **(r)**, SW620 **(s)**, and H1299 **(t)** cells transfected with the indicated plasmids expressing backbone, IRF3 or IRF3 mutants treated as in **(q)**. **(u)** Real time qPCR analysis for the Wnt target and associated genes in IRF3$^{+/+}$ and IRF3$^{-/-}$ H1299 cells transfected with the indicated plasmids expressing backbone, IRF3 or IRF3 mutants. **(v)** TOPflash-relative luciferase activity analysis of β-catenin-S33A co-transfected with IRF3 or its mutants in HEK293T. **(w)** Immunoblot analysis of p-IRF3 in flag-GFP and flag-RIG-I-N transfected HEK293T cells. **(x)** TOPflash-relative luciferase activity analysis of β-catenin-S33A co-transfected with IRF3 or its mutants in IRF3-knockout HCT116 cell line. Each symbol represents an individual mouse **(l, n-o)**. $^*P< 0.05$; $^{**}P< 0.01$; $^{***}P< 0.001$; NS, not statistically significant by two tailed t-test. Data represent two (**m-o**) or three (**a-l, p-x**) independent experiments and are presented as mean± s.e.m. in **a-x.**

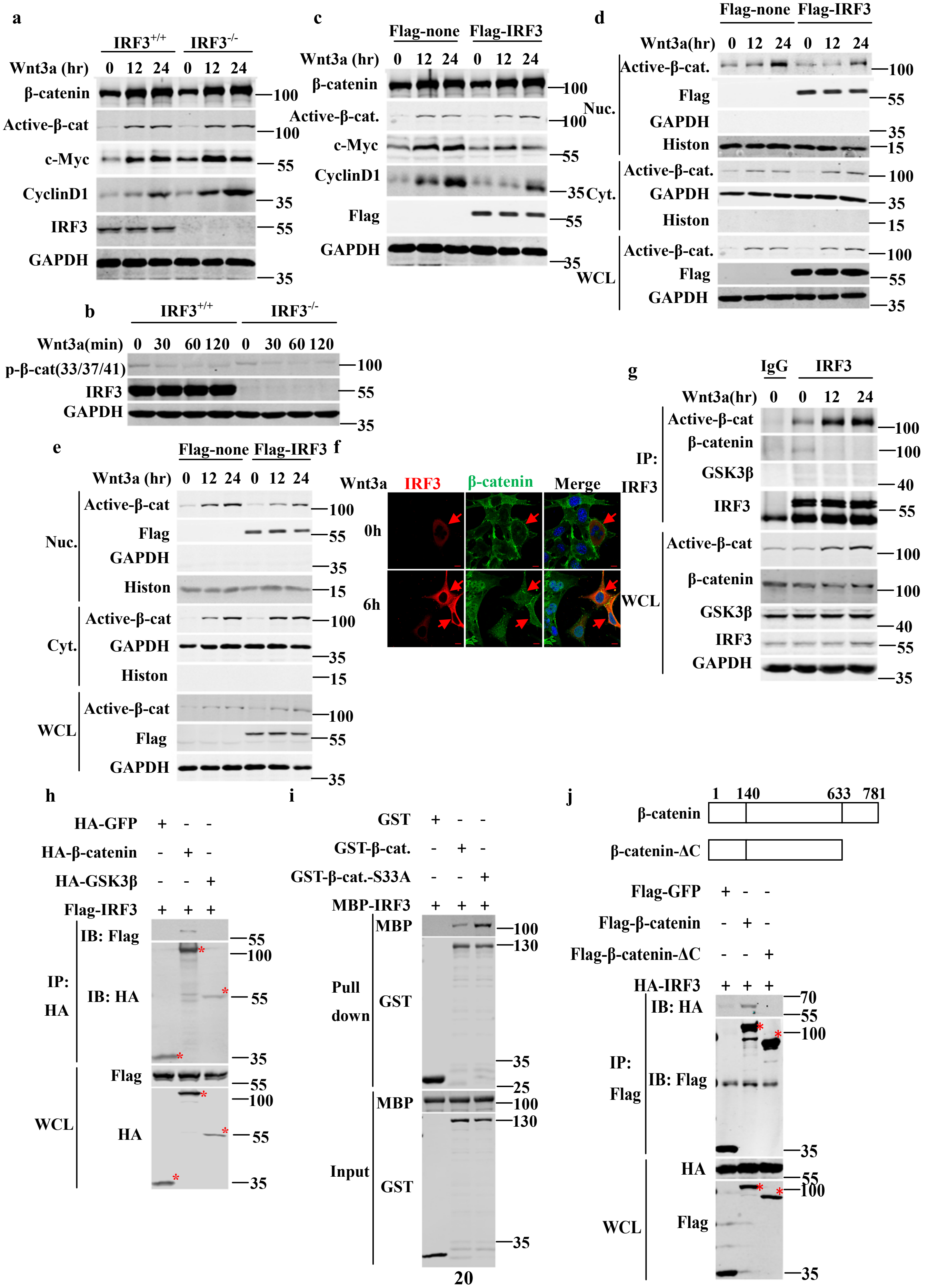
a
IRF3+/+
IRF3-/-
Wnt3a (hr)
0 12 24 0 12 24
β-catenin
Active-β-cat
c-Myc
CyclinD1
IRF3
GAPDH
100
100
55
35
55
35
b
Wnt3a(min)
0 30 60 120 0 30 60 120
p-β-cat(33/37/41)
IRF3
GAPDH
c
Flag-none
Flag-IRF3
Active-β-cat.
Flag
d
Nuc.
Cyt.
WCL
Histon
e
f
Wnt3a
IRF3
β-catenin
Merge
0h
6h
g
IgG
IRF3
IP:
IRF3
GSK3β
h
HA-GFP
HA-β-catenin
HA-GSK3β
Flag-IRF3
IB: Flag
IB: HA
IP:
HA
i
GST
GST-β-cat.
GST-β-cat.-S33A
MBP-IRF3
MBP
Pull
down
Input
j
1 140 633 781
β-catenin
β-catenin-ΔC
Flag-GFP
Flag-β-catenin
Flag-β-catenin-ΔC
HA-IRF3
IP:
Flag

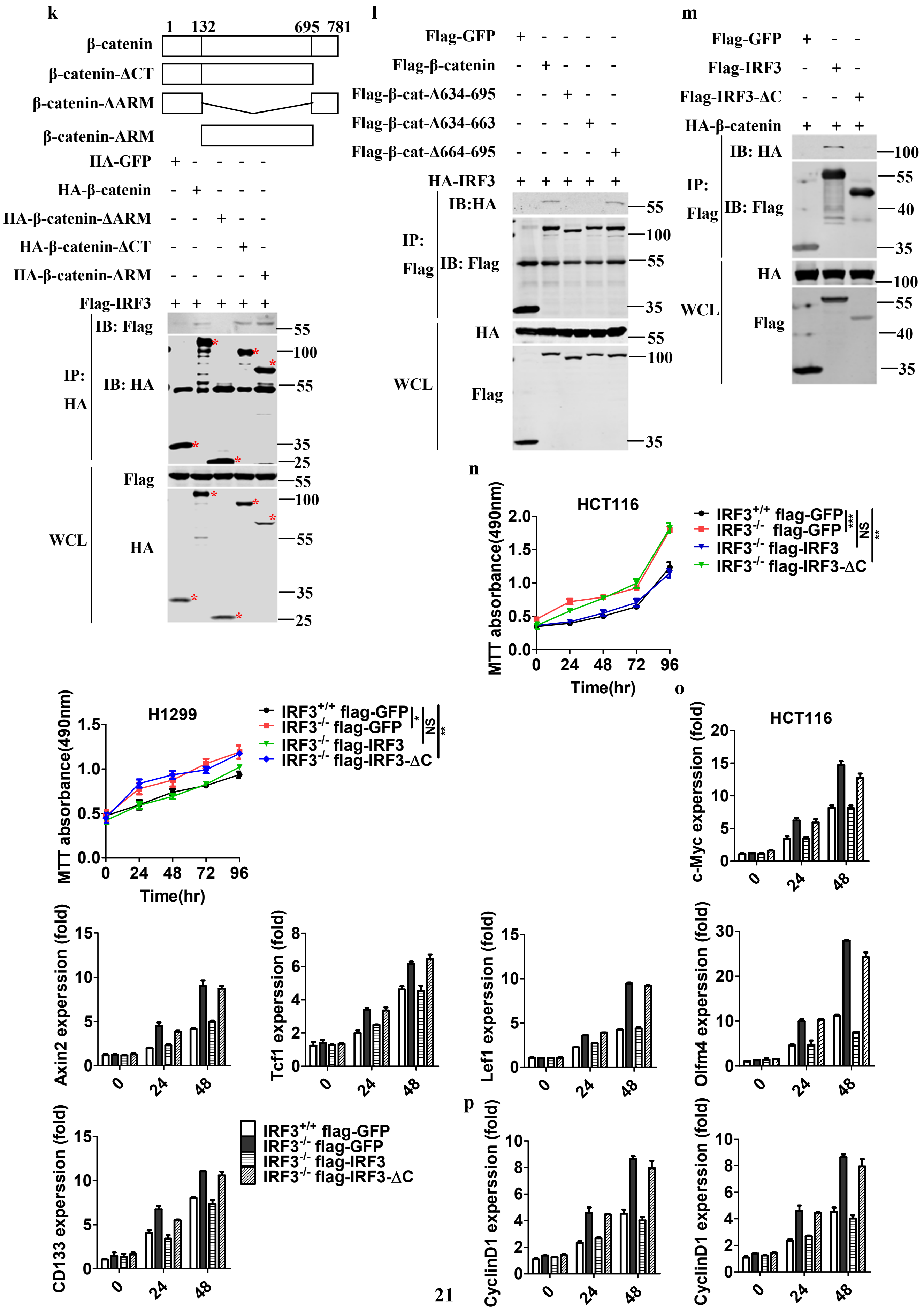
k
β-catenin
1 132 695 781
β-catenin-ΔCT
β-catenin-ΔARM
β-catenin-ARM
HA-GFP
HA-β-catenin
HA-β-catenin-ΔARM
HA-β-catenin-ΔCT
HA-β-catenin-ARM
Flag-IRF3
IP: HA
IB: Flag
IB: HA
WCL
Flag
HA
l
Flag-GFP
Flag-β-catenin
Flag-β-cat-Δ634-695
Flag-β-cat-Δ634-663
Flag-β-cat-Δ664-695
HA-IRF3
IP: Flag
IB:HA
IB: Flag
WCL
HA
Flag
m
Flag-GFP
Flag-IRF3
Flag-IRF3-ΔC
HA-β-catenin
IP: Flag
IB: HA
IB: Flag
WCL
HA
Flag
n
HCT116
MTT absorbance(490nm)
Time(hr)
IRF3+/+ flag-GFP
IRF3-/- flag-GFP
IRF3-/- flag-IRF3
IRF3-/- flag-IRF3-ΔC
***
NS
**
H1299
*
NS
**
o
HCT116
c-Myc expression (fold)
Axin2 expression (fold)
Tcf1 expression (fold)
Lef1 expression (fold)
Olfm4 expression (fold)
CD133 expression (fold)
p
CyclinD1 expression (fold)
CyclinD1 expression (fold)

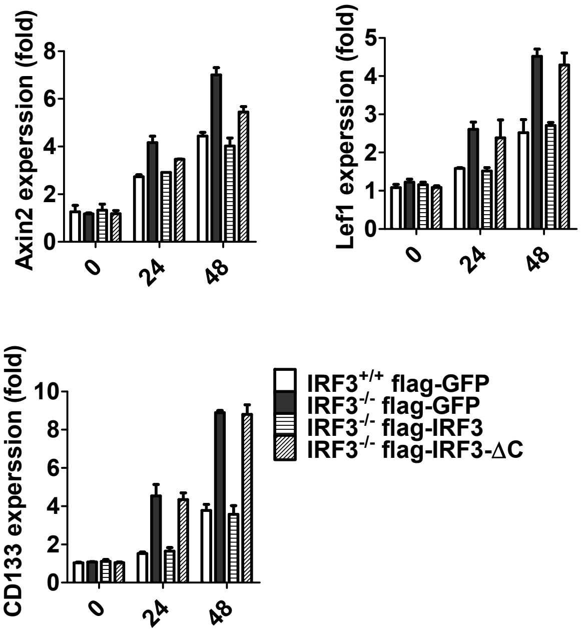
Axin2 experssion (fold)
0
2
4
6
8
0
24
48
Lef1 experssion (fold)
0
1
2
3
4
5
0
24
48
Tcf1 experssion (fold)
0
2
4
6
8
0
24
48
CD133 experssion (fold)
0
2
4
6
8
10
0
24
48
IRF3+/+ flag-GFP
IRF3-/- flag-GFP
IRF3-/- flag-IRF3
IRF3-/- flag-IRF3-ΔC

q

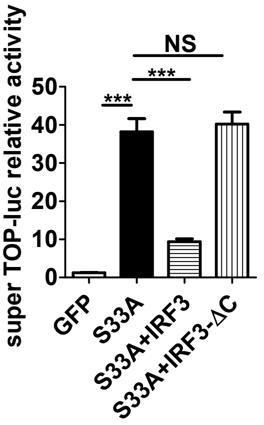
super TOP-luc relative activity
0
10
20
30
40
50
NS
***
***
GFP
S33A
S33A+IRF3
S33A+IRF3-ΔC

**Supplementary Figure 5. IRF3 binds to the ARM domain of β-catenin to inhibit its nucleus translocation. Related to Figure 5. (a-b)** Immunoblot analysis of Wnt signal pathway in IRF3 deficient HCT116 cells with wnt3a-conditioned medium treatment. **(c)** Immunoblot analysis of Wnt signal pathway in IRF3 overexpressed HCT116 cells with wnt3a-conditioned medium treatment. **(d-e)** Nucleocytoplasmic separation and immunoblot analysis of active-β-catenin in IRF3 overexpressed HCT116 **(d)** and H1299 **(e)** cells with wnt3a-conditioned medium treatment. **(f)** Immunofluorescence analysis of β-catenin cellular localization with anti-β-catenin (Green) and anti-IRF3 (Red) antibody in IRF3 overexpressed HEK293 cells with or without wnt3a-conditioned medium treatment. The red arrowheads indicate IRF3-overexpressed cells. Scale bars, 10 μm. **(g)** Immunoblot analysis of the endogenous interaction between active-β-catenin, β-catenin or GSK3β and IRF3 with anti-IRF3 immunoprecipitates in H1299 cell line extracts after treated with wnt3a-conditioned medium. **(h)** Immunoblot analysis of the interaction between GSK3β and IRF3 with anti-HA immunoprecipitates in HEK293T cell line. **(i)** GST pull-down analysis for the interaction between GST-β-catenin or GST-β-catenin-S33A with MBP-IRF3. **(j)** Immunoblot analysis for the interaction between β-catenin or β-catenin-ΔC and IRF3 with anti-FLAG immunoprecipitates in HEK293T cells. **(k)** Immunoblot analysis for the interaction between β-catenin, β-catenin-ΔARM, β-catenin-ΔCT or β-catenin-ARM and IRF3 with anti-HA immunoprecipitates of HEK293T cells. **(l)** Immunoblot analysis for the interaction between β-catenin, Δ634-695, Δ634-663 or Δ664-695 and IRF3 with anti-Flag immunoprecipitates of HEK293T cells. **(m)** Immunoblot analysis for the interaction between IRF3 or IRF3-ΔC and β-catenin with anti-HA immunoprecipitates of HEK293T cells. **(n)** Proliferation of IRF3$^{+/+}$ and IRF3$^{-/-}$ cells rescued with flag-IRF3 or flag-IRF3-ΔC. **(o-p)** Real time qPCR analysis for the Wnt target and associated genes expression in IRF3$^{+/+}$ and IRF3$^{-/-}$ HCT116 and H1299 cells rescued with flag-IRF3 or flag-IRF3-ΔC. **(q)** TOPflash-relative luciferase activity analysis for β-catenin-S33A co-transfected with IRF3 or IRF3-ΔC in HEK293T cells. *$P< 0.05$; **$P< 0.01$; ***$P< 0.001$; NS, not statistically significant by two tailed t-test **(n-q)**. Data represent three independent experiments **(a-q)** and are presented as mean± s.e.m. in **n-q**.

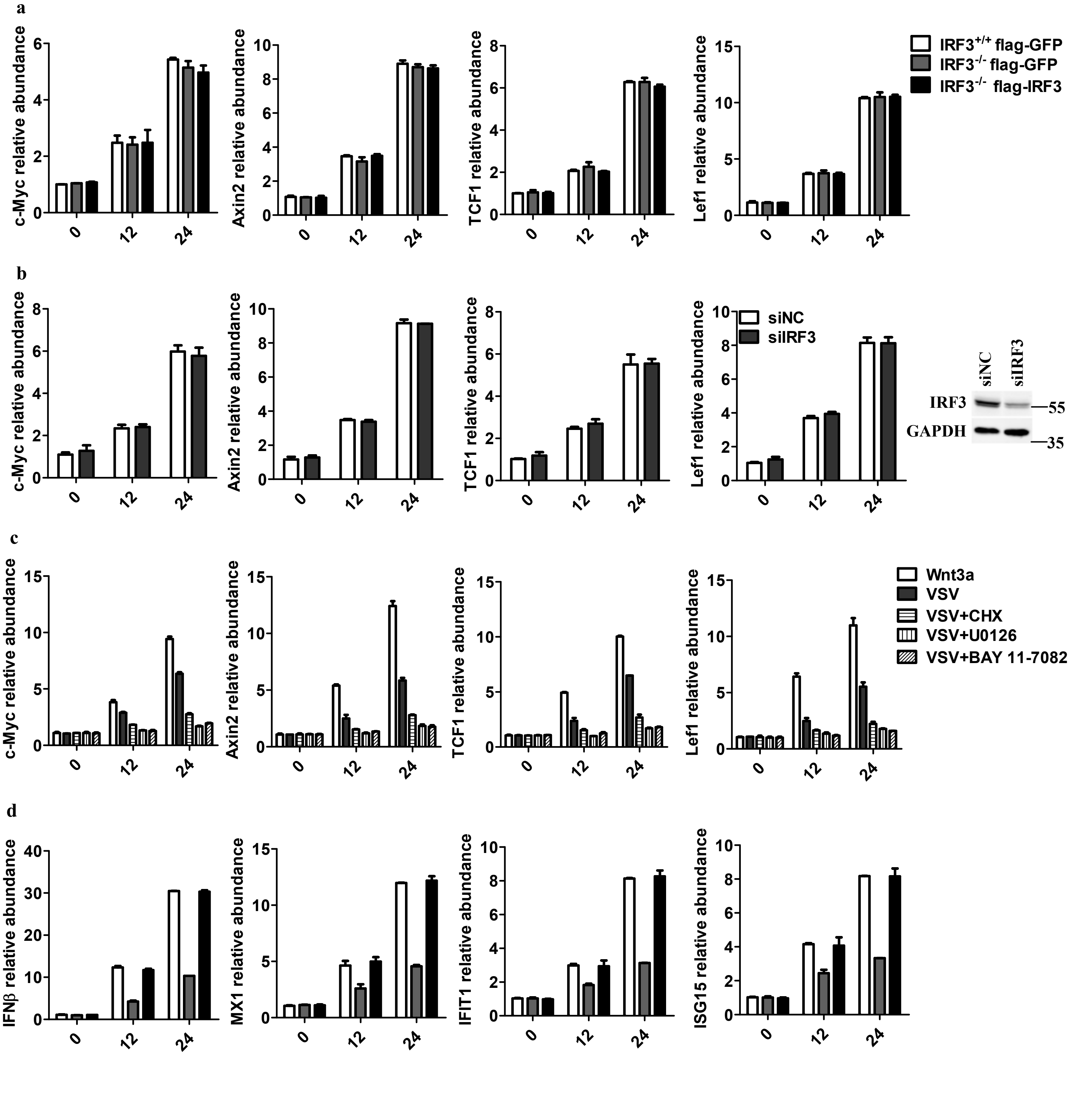
a
c-Myc relative abundance
Axin2 relative abundance
TCF1 relative abundance
Lef1 relative abundance
IRF3+/+ flag-GFP
IRF3-/- flag-GFP
IRF3-/- flag-IRF3
0
12
24
b
siNC
siIRF3
IRF3
GAPDH
55
35
c
Wnt3a
VSV
VSV+CHX
VSV+U0126
VSV+BAY 11-7082
d
IFNβ relative abundance
MX1 relative abundance
IFIT1 relative abundance
ISG15 relative abundance

e

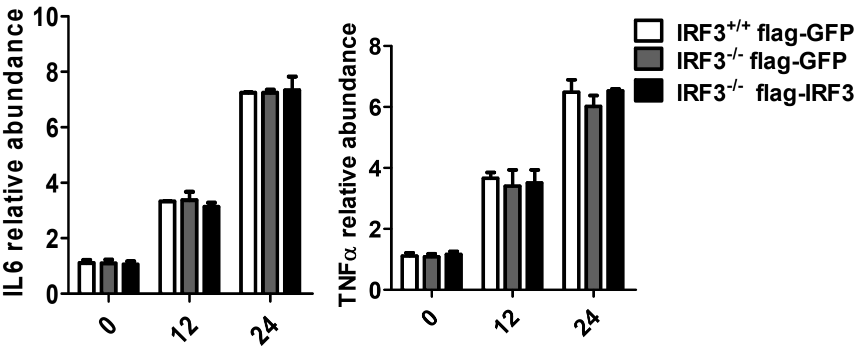
IL6 relative abundance
TNFα relative abundance
0
2
4
6
8
10
0
12
24
IRF3+/+ flag-GFP
IRF3-/- flag-GFP
IRF3-/- flag-IRF3

f

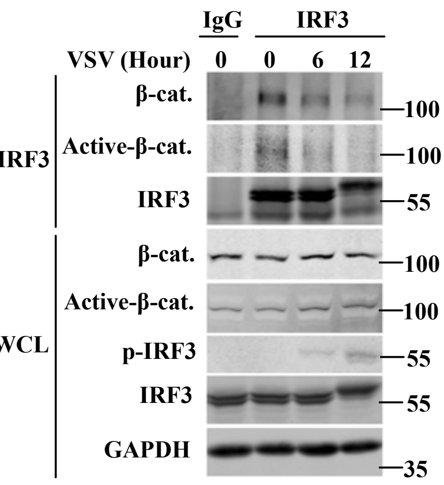
IgG
IRF3
VSV (Hour)
0
0
6
12
IP: IRF3
β-cat.
Active-β-cat.
IRF3
WCL
β-cat.
Active-β-cat.
p-IRF3
IRF3
GAPDH
100
100
55
100
100
55
55
35

g

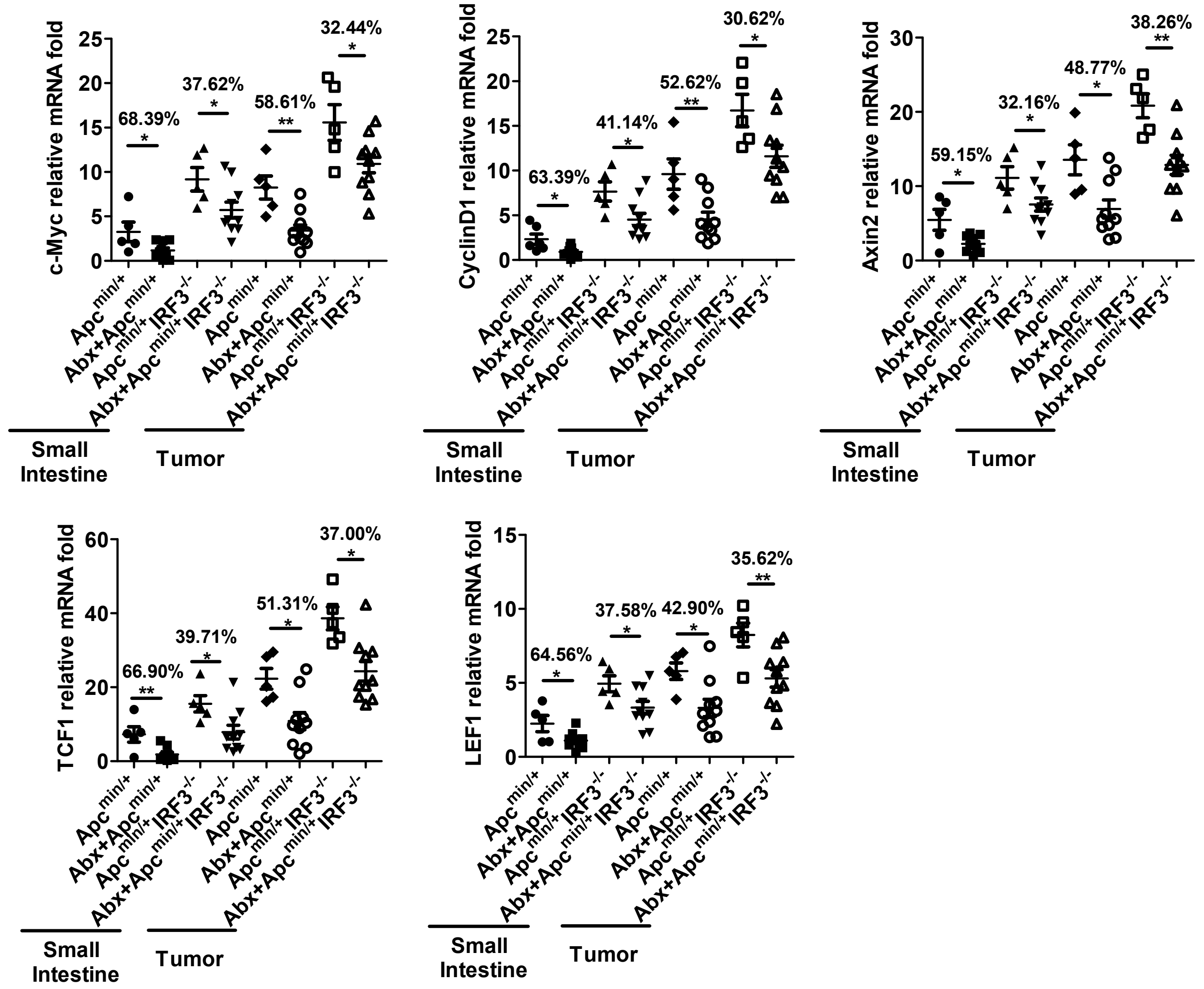
c-Myc relative mRNA fold
68.39%
37.62%
58.61%
32.44%
CyclinD1 relative mRNA fold
63.39%
41.14%
52.62%
30.62%
Axin2 relative mRNA fold
59.15%
32.16%
48.77%
38.26%
TCF1 relative mRNA fold
66.90%
39.71%
51.31%
37.00%
LEF1 relative mRNA fold
64.56%
37.58%
42.90%
35.62%
Apcmin/+
Abx+Apcmin/+
Apcmin/+ IRF3-/-
Abx+Apcmin/+ IRF3-/-
Small Intestine
Tumor

**h**

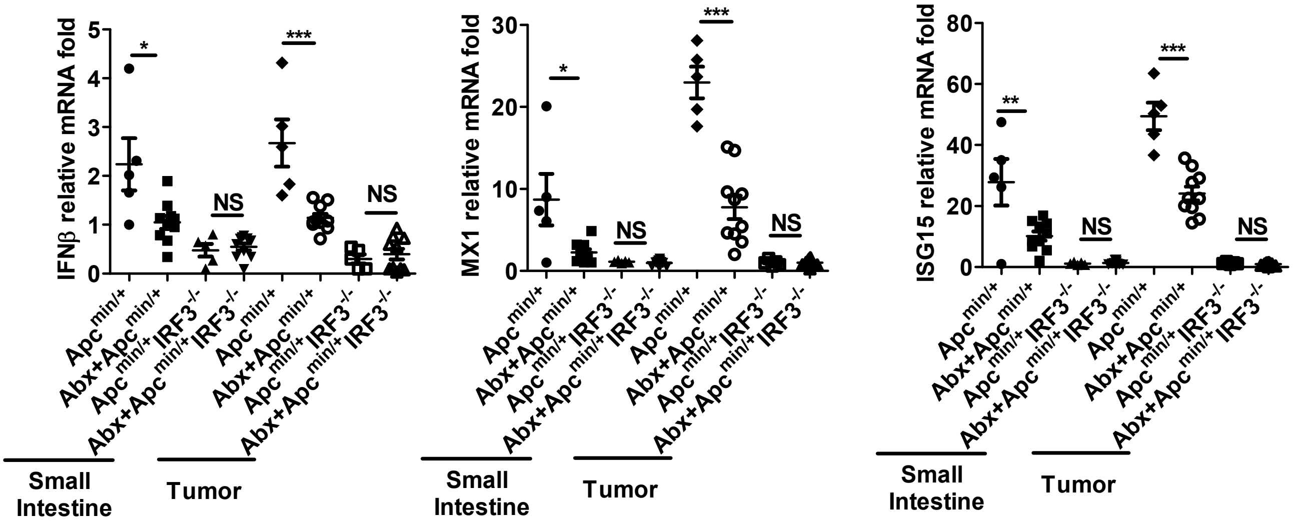
IFNβ relative mRNA fold
MX1 relative mRNA fold
ISG15 relative mRNA fold
*
**
***
NS
Apc min/+
Abx+Apc min/+
Apc min/+ IRF3-/-
Abx+Apc min/+ IRF3-/-
Small Intestine
Tumor

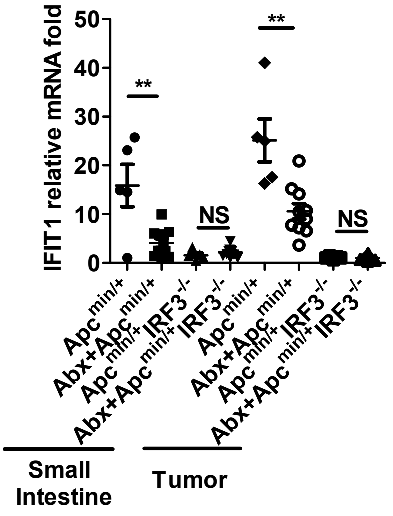
IFIT1 relative mRNA fold
**
NS
Apc min/+
Abx+Apc min/+
Apc min/+ IRF3-/-
Abx+Apc min/+ IRF3-/-
Small Intestine
Tumor

**i**

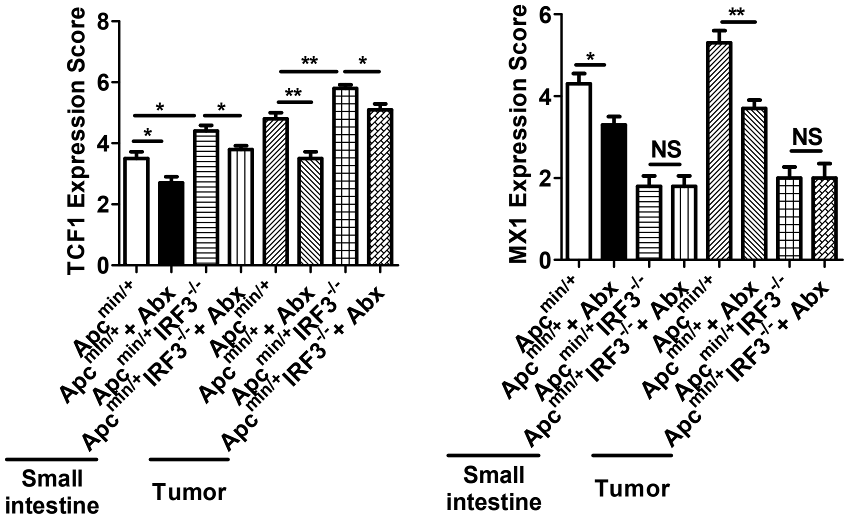
TCF1 Expression Score
MX1 Expression Score
*
**
NS
Apc min/+
Apc min/+ + Abx
Apc min/+ IRF3-/-
Apc min/+ IRF3-/- + Abx
Small intestine
Tumor

**Supplementary Figure 6. Activation of IRF3 by PRR facilitates Wnt signaling. Related to Figure 6. (a)** Real-time qPCR analysis for the Wnt target genes in rescued HCT116 cas9 cells stimulated with VSV. **(b)** Real-time qPCR analysis for the Wnt target genes in IRF3-knockdown HCT116 cells stimulated with VSV. Immunoblot analysis for IRF3 in siNC and siIRF3 HCT116 cells. **(c)** Real-time qPCR analysis for the Wnt target genes in HCT116 cells stimulated with CHX/U0126 (MAPK signaling inhibitor)/BAY 11-7082 (NF-κB signaling inhibitor) and VSV. **(d)** Real-time qPCR analysis for IFNβ and ISGs (MX1, IFIT1, ISG15) in rescued HCT116 cas9 cells stimulated with VSV. **(e)** Real-time qPCR analysis for IL6 and TNFα in rescued HCT116 cas9 cells stimulated with VSV. **(f)** Immunoblot analysis for the endogenous interaction between active-β-catenin or β-catenin and IRF3 with anti-IRF3 immunoprecipitates in H1299 cell line extracts treated with VSV. **(g)** Real-time qPCR analysis for the Wnt target and associated genes in the small intestine and tumors from $Apc^{min/+}$ and $Apc^{min/+}$ $IRF3^{-/-}$ mice with or without Abx treatment (n=5, n=10, n=5, n=10), the number indicates the decreased rate induced by Abx treatment. **(h)** Real-time qPCR analysis for the IFNβ and ISGs (MX1, ISG15, IFIT1) in the small intestine and tumors from $Apc^{min/+}$ and $Apc^{min/+}$ $IRF3^{-/-}$ mice with or without Abx treatment (n=5, n=10, n=5, n=10). **(i)** Quantification of the expression score of TCF1 and MX1 in $Apc^{min/+}$ and $Apc^{min/+}$ $IRF3^{-/-}$ mice with or without Abx treatment (n=5 mice/group). Each symbol represents one mouse (**g-h**). *P< 0.05; **P< 0.01; ***P< 0.001; NS, not statistically significant by two-tailed *t*-test (**a-e, g-i** ). Data are from two (**g-i)** or three (**a-f)** independent experiments and are presented as mean± s.e.m. in **a-i.**

a

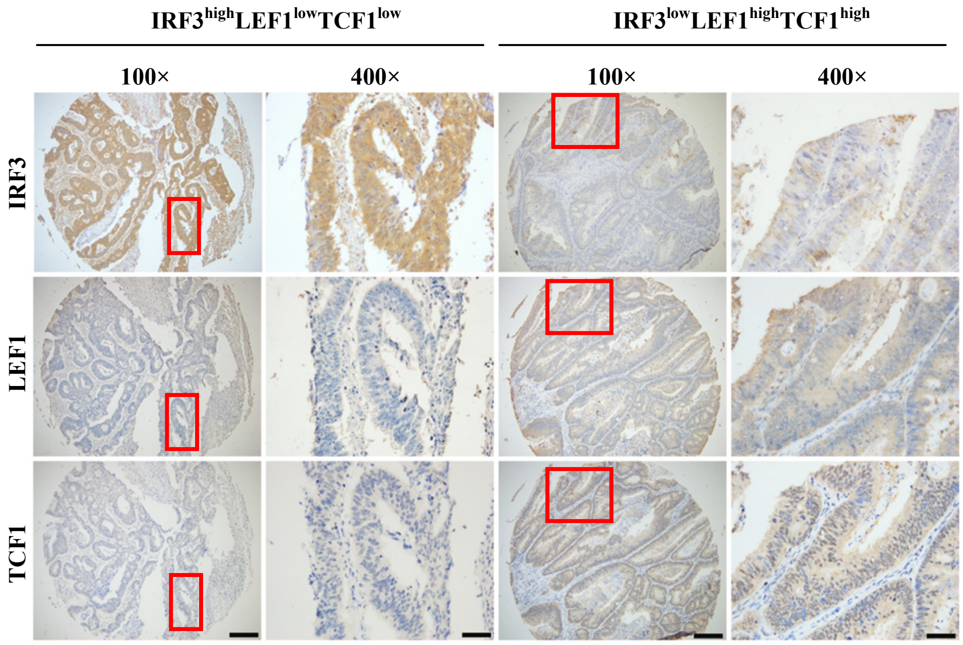
IRF3$^{high}$LEF1$^{low}$TCF1$^{low}$
IRF3$^{low}$LEF1$^{high}$TCF1$^{high}$
100×
400×
100×
400×
IRF3
LEF1
TCF1

b

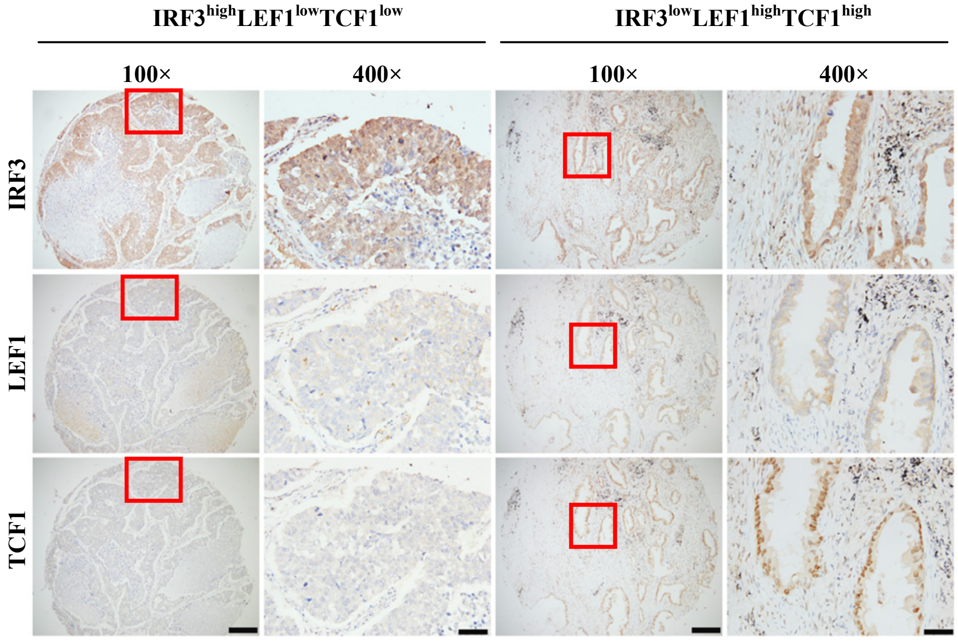
IRF3$^{high}$LEF1$^{low}$TCF1$^{low}$
IRF3$^{low}$LEF1$^{high}$TCF1$^{high}$
100×
400×
100×
400×
IRF3
LEF1
TCF1

c

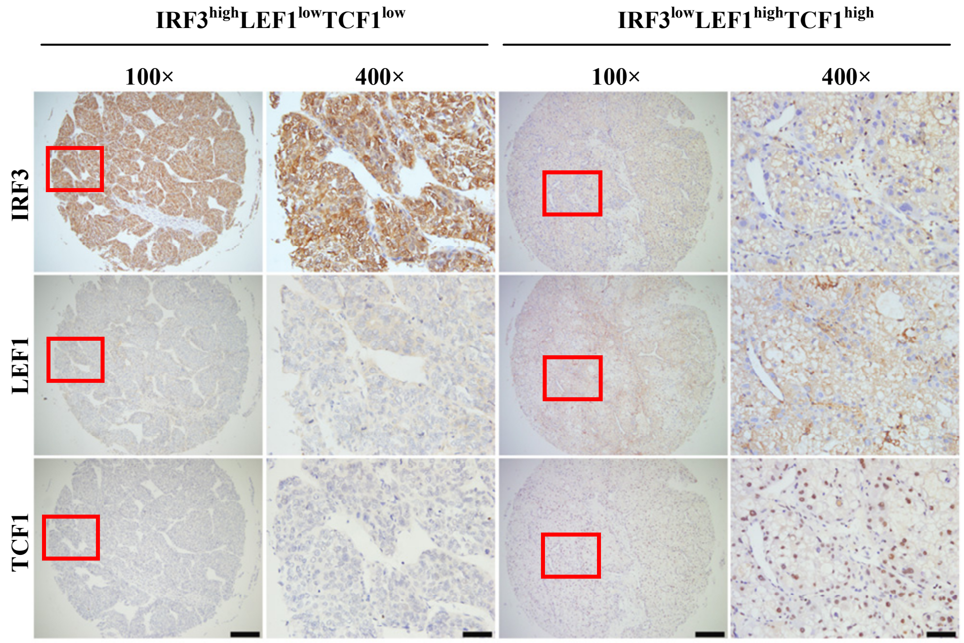


**Supplementary Figure 7. IRF3 expression is increased in human cancer. Related to Figure 7.** **(a-c)** Immunohistochemical analysis for IRF3 (upper), LEF1 (middle) and TCF1 (down) expression in tumors of one human CRC **(a)**, lung adenocarcinoma **(b)** and hepatocellular carcinoma **(c)** specimen. 100×, scale bars, 200 µm; 400×, scale bars, 50 µm.

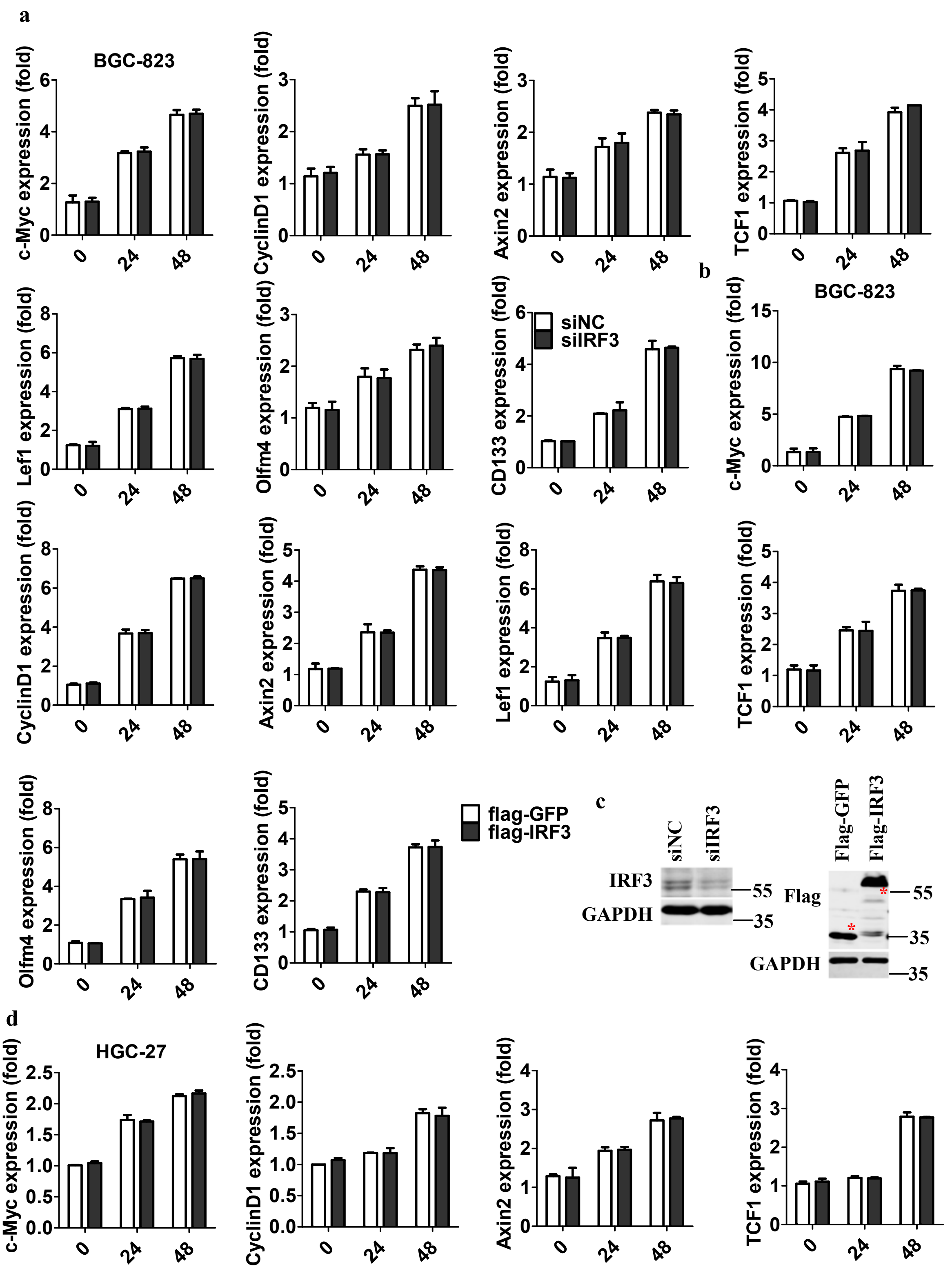
a
BGC-823
c-Myc expression (fold)
CyclinD1 expression (fold)
Axin2 expression (fold)
TCF1 expression (fold)
Lef1 expression (fold)
Olfm4 expression (fold)
CD133 expression (fold)
siNC
siIRF3
b
BGC-823
flag-GFP
flag-IRF3
c
siNC
siIRF3
IRF3
GAPDH
Flag-GFP
Flag-IRF3
Flag
55
35
d
HGC-27
0
24
48

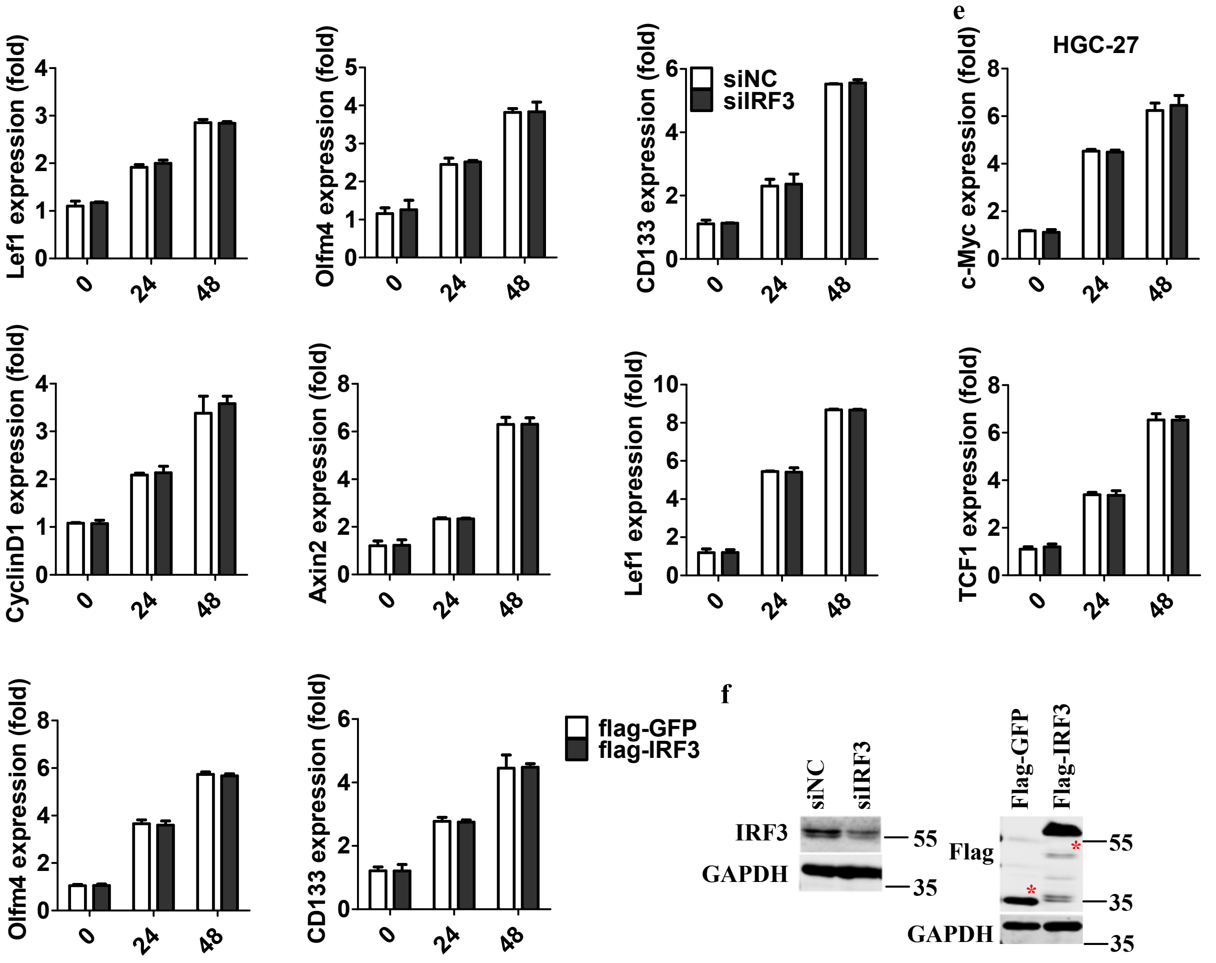
e
HGC-27
siNC
siIRF3
Lef1 expression (fold)
Olfm4 expression (fold)
CD133 expression (fold)
c-Myc expression (fold)
CyclinD1 expression (fold)
Axin2 expression (fold)
Lef1 expression (fold)
TCF1 expression (fold)
Olfm4 expression (fold)
CD133 expression (fold)
flag-GFP
flag-IRF3
0
24
48
f
siNC
siIRF3
IRF3
GAPDH
55
35
Flag-GFP
Flag-IRF3
Flag
GAPDH
55
35
35

**Supplementary Figure 8. IRF3 has no effect on Wnt/β-catenin pathway in BGC-823 and HGC-27 cell lines. (a)** Real time qPCR analysis for the Wnt target and associated genes in the siIRF3 or siNC transfected BGC-823 cells with wnt3a-conditioned medium treatment. **(b)** Real time qPCR analysis for the Wnt target and associated genes in BGC-823 cells transfected with the indicated plasmids expressing backbone or IRF3 treated as in **(a)**. **(c)** Immunoblot analysis of the down-regulation (left) and overexpression (right) of IRF3 in transfected BGC-823 cells. **(d)** Real time qPCR analysis for the Wnt target and associated genes in the siIRF3 or siNC transfected HGC-27 cells with wnt3a-conditioned medium treatment. **(e)** Real time qPCR analysis for the Wnt target and associated genes in HGC-27 cells transfected with the indicated plasmids expressing backbone or IRF3 treated as in **(d)**. **(f)** Immunoblot analysis of the down-regulation (left) and overexpression (right) of IRF3 in transfected HGC-27 cells. Data represent three (**a-f**) independent experiments and are presented as mean± s.e.m. in **a-b** and **d-e.**

**Supplementary Table 1. Tumor number decreasement with antibiotics in Apc$^{min/+}$ and Apc$^{min/+}$IRF3$^{-/-}$ mice in two independent experiments**

| Mice | Average tumor number | | Decreased rate (%) | | p-value |
|---|---|---|---|---|---|
| | 1$^{st}$ | 2$^{nd}$ | 1$^{st}$ | 2$^{nd}$ | |
| **Apc$^{min/+}$** | 8.1±4.3 | 8.8±3.7 | 67.45 | 70.45 | 0.017 |
| **Apc$^{min/+}$+ Abx** | 2.6±2.3 | 2.6±2.4 | | | |
| **Apc$^{min/+}$IRF3$^{-/-}$** | 22.9±15.3 | 35.0±15.8 | 55.02 | 55.71 | |
| **Apc$^{min/+}$IRF3$^{-/-}$ +Abx** | 10.3±6.9 | 15.5±7.5 | | | |

$p<0.05$ shows statistical significant by two-way *t-test*.

## 2. Supplementary methods

### Reagents and Plasmids

Azoxymethane (AOM) and M2 (anti-Flag) beads were purchased from Sigma Aldrich; DSS was purchased from MP Biomedicals (M.W. =36,000-50,000). ICG-001 was purchased from Med Chem Express (MCE). G007-LK was purchased from Good Laboratory Practice bioscience (GLPBIO). Akt (1:1000), p-Akt (1:1000), p-Stat3 (1:1000), β-catenin (1:1000), active-β-catenin (1:1000), p-β-catenin (1:1000), p-JNK (1:1000), JNK (1:1000), p-ERK1/2 (1:1000), ERK1/2 (1:1000), p-p38 (1:1000), p38 (1:1000), cyclinD1 (1:1000), c-Myc (1:1000), Phospho-IRF3 (Ser386) (1:1000), GADPH (1:2000), Histone H3 (1:2000), β-Tubulin Antibody (1:2000), TCF1/TCF7 (1:100), HA-Tag (1:4000), STING (D2P2F, 1:1000) and MAVS (D5A9E, 1:1000) antibodies were purchased from Cell Signaling Technology. Lysozyme antibody(1:500) was purchased from Abcam. IRF3 (1:1000) and LEF1 (1:50) antibodies were purchased from Proteintech. Anti-Flag (1:1000) and His (1:1000) antibodies were purchased from Abmart. Anti-GST (1:1000) and anti-MBP (1:1000) were purchased from HUABIO. DAPI was purchased from Life Technology. GST beads and Lipofectamine$^{@}$ 3000 reagent were purchased from Thermo Scientific. PEI was purchased from Polyscience, and INTERFERin$^{@}$ was purchased from Polyplus Transfection. Scramble siRNA and IRF3 target siRNA were purchased from Genepharma (Shanghai, China). HA-IRF3 plasmid was cloned from the HCT116 cell line cDNA and constructed in the pcDNA3.1-HA-His vector, and flag-IRF3, flag-IRF3-ΔnDB, flag-IRF3-ΔNLS, and flag-IRF3-5D were subcloned into the pcDNA3.1-flag-His vector. TOPflash plasmid and HA-β-catenin were kind gifts from Wand's Lab (Tongji University). Flag-IRF3 and flag-β-catenin deletion mutants were subcloned into the pcDNA3.1-Flag-His vector; GST-β-catenin, GST-β-catenin-ARM, GST-β-catenin-Δ632-663, GST-β-catenin-S33A and MBP-IRF3 were subcloned into the pGEX-4T1-GST-10His vector and pGEX-4T1-MBP-10His vector respectively.

### Bone Marrow Chimeras

Briefly, recipient mice underwent sub-lethal dose of γ-ray irradiation (8.5Gly) to kill the bone marrow cell and six hours post-irradiation, IRF3$^{+/+}$ and IRF3$^{-/-}$ recipients were received 100 μl fresh IRF3$^{+/+}$ and IRF3$^{-/-}$ bone marrow cells with the concentration of $1\times10^{8}$/ml respectively, which were IRF3$^{+/+}$→IRF3$^{+/+}$, IRF3$^{-/-}$→IRF3$^{+/+}$ and IRF3$^{+/+}$→IRF3$^{-/-}$ groups. 8 weeks after bone marrow transplantation, mice's blood were collected and determined with a IRF3 genotyping analysis to exclude failure mice, then injected with AOM (10mg/kg) and fed with 2.5% DSS for indicated time to induce colorectal cancer.

### Immunohistochemistry, TUNEL assay and Human Tissue Microarray

Immunohistochemistry and the TUNEL assay (one-step TUNEL apoptosis assay kit (Roche)) were performed for mouse colonic sections by the Histomorphology Platform, Zhejiang University, with the standard protocol performed according to the manufacturer's instructions. HEK293 overexpressing flag-IRF3 cells cultured on cover slips were fixed for 15 mins with 4% PFA and permeabilized for 10 min at room temperature with 0.1% Triton X-100 in 1×PBS. After incubation for blockade of nonspecific binding for 30 mins, rabbit anti-IRF3 antibody (1:50, Proteintech, #11312-1-AP) and mouse anti-β-catenin (1:200, CST, #2677) were added and incubated for overnight at 4°C. Samples were further stained with goat anti-mouse Alexa Fluor 488–conjugated and goat anti-rabbit Alexa Fluor 594–conjugated secondary antibodies (Thermo Fisher Scientific) for 1 h at room temperature. Images were acquired on an Olympus IX81-FV1000 fluorescence microscope (Olympus). Immunohistochemistry staining of the tissue microarray (TMA) of colorectal cancer (115 cases, Sir Run Run Shaw Hospital School of Medicine, Zhejiang University) was performed by Servicebio. According to the histopathological characteristics in clinicopathological features of the colorectal cancer patients, we collected the clinical samples which were classified as adenocarcinomas-NOS (Not Otherwise Specified). CRC variants (such as mucinous adenocarcinoma, signet ring cell carcinoma, serrated adenocarcinoma and so on) were not included in our study [1]. Lung adenocarcinoma and hepatocellular carcinoma TMA were purchased from Shanghai outdo biotech co., LTD, and the immunohistochemistry staining of them were also performed by Shanghai outdo biotech co., LTD. The staining extent score was on a scale of 0-3, corresponding to the percentage of immunoreactive tumor cells (0%-10%, 11%-25%, 26%-75% and 76%-100%, respectively) and the staining intensity (negative, score = 0; weak, score = 1; strong, score = 2; very strong, score =3). A score ranging from 0-3 was calculated by adding the staining extent score with the intensity score, resulting in a low (< median) level or a high (≥ median) level value for each specimen. The number of Ki67- or BrdU-positive cells per crypt in each animal was counted (at least 18–20 crypts per mouse). To visualize Paneth cells and goblet cells, ileum and colon tissues were stained with anti-lysozyme antibody (1:1000, Abcam, #ab108508) and PAS and Alcian blue. The number of Paneth cells and goblet cells per crypt were enumerated.

**Gut Microbiome Analysis**

Feces were removed from the distal part of the colon on day 90 after AOM/DSS treatment. Genomic DNA from feces were extracted by QiAMP DNA stool Mini Kit (QIAGEN) and used for 16S rDNA gene sequencing. Sequencing libraries were generated using TruSeq® DNA PCR-Free Sample Preparation Kit (Illumina, USA) following manufacturer's recommendations and index codes were added. The library quality was assessed on the Qubit@ 2.0 Fluorometer (Thermo Scientific) and Agilent Bioanalyzer 2100 system. At last, the library was sequenced on an Illumina HiSeq 2500 platform and 250 bp paired-end reads were generated. The library construction and sequencing was performed at Novogene (Beijing, China), and the data were also analyzed by Novogene.

**Co-housing experiments**

For co-housing experiments, equal numbers of 4-week-old $IRF3^{+/+}$ and $IRF3^{-/-}$ mice were co-housed in the same cage for 4 weeks before injection of AOM (10mg/kg) and remained co-housed over the course of the AOM/DSS model.

**Tumor graft model**

Tumor were excised from colon from $IRF3^{+/+}$ and $IRF3^{-/-}$ mice and incubated in cold 1×PBS (137 mM NaCl, 2.7 mM KCl, 10 mM $Na_2HPO_4$, 2 mM $KH_2PO_4$, 10 µg/ml Amphotericin B, 15µg/ml gentamicin, 10 U/ml penicillin, 1 µg/ml streptomycin). The tumor were cut into 2 × 2 × 3 $mm^3$ pieces by a sterile

surgical scissors, and then washed three times with the above 1×PBS. After the nude mouse was anesthetized with chloroform, use 75% alcohol to disinfect the skin of the nude mouse's back and abdomen, and then cut a small hole near the side of the nude mouse with a surgical knife. The prepared tumor fragments were placed into the small hole, and then it was sutured with a surgical line. Tumor growth was measured about once 10-20 days with a caliper. The estimated tumor volume (V) was calculated by the formula $V = W \times L^2 \times 0.5$, where W represents the largest tumor diameter in centimeters and L represents the next largest tumor diameter.

**RNA-seq analysis**

The RNA for the RNA-seq experiment was extracted from colon and tumor tissues of $IRF3^{+/+}$ or $IRF3^{-/-}$ mice on day 90 of the AOM/DSS model. Total RNA was isolated using RNeasy mini kit (Qiagen, Germany). Strand-specific libraries were prepared using the TruSeq® Stranded Total RNA Sample Preparation kit (Illumina, USA) following the manufacturer's instructions. Briefly, ribosomal RNA was removed from total RNA using Ribo-Zero rRNA removal beads. Following purification, the mRNA is fragmented into small pieces using divalent cations under 94℃ for 8 min. The cleaved RNA fragments are copied into first strand cDNA using reverse transcriptase and random primers. This is followed by second strand cDNA synthesis using DNA Polymerase I and RNase H. These cDNA fragments then go through an end repair process, the addition of a single 'A' base, and then ligation of the adapters. The products are then purified and enriched with PCR to create the final cDNA library. Purified libraries were quantified by Qubit® 2.0 Fluorometer (Life Technologies, USA) and validated by Agilent 2100 bioanalyzer (Agilent Technologies, USA) to confirm the insert size and calculate the mole concentration. Cluster was generated by cBot with the library diluted to 10 pM and then were sequenced on the Illumina HiSeq 2500 (Illumina, USA).

**RNA in situ hybridization (ISH) in normal crypts**

Fourteen to 4 μm paraffin sections were used. Probe concentrations were 100 ng/100 μl hybridization buffer. Hybridization incubation was at 65°C for 18–20 h. After hybridization, sections were treated with 10 μg/ml RNase A to eliminate nonspecific binding. Slices were incubated with DAPI solution in dark for 8 minutes, then sealed with water anti-fluorescence quenching sealing tablets after washing. Collect images under the Nikon positive fluorescence microscope: DAPI glows blue by UV excitation wavelength 330-380 nm and emission wavelength 420 nm; FAM glows green by excitation wavelength 465-495 nm and emission wavelength 515-555 nm; CY3 glows red by excitation wavelength 510-560 nm and emission wavelength 590 nm. And the probe sequences for Lgr5 and Axin2 were as follows: Lgr5 probe: 5'-CY3-GATGTGGTTGGCATCTAGGCGCAGGGAT-CY3-3'; AXIN2 probe: 5'-FAM-TCGTCGGTCAGTGCGTCGCTGGATAA-FAM-3'.

**Real time qPCR**

Total RNA of cell lines and tissues was extracted using TRIzol (Invitrogen) according to the manufacturer's instructions. RNA was reverse transcribed using M-MLV reverse transcriptase (Takara). The quantification of gene transcripts was performed by real-time PCR using SYBR Green master mix (Vazyme) and the 480П real-time PCR system (Roche). β-actin served as an internal control in mice tissue, and GADPH served as an internal control in human tumor cell lines. The specific primers for individual genes are in shown as follows: **Supplementary Table 3.**

**Immunoblot analysis and immunoprecipitation**

Colon and tumor tissues were lysed with cell lysis buffer (Cell Signaling Technology) on ice for 30mins, and then were centrifuged at 12,000 rpm for 15mins. The supernatant protein concentration was determined by the BCA protein assay kit (Thermo). Cell lysates (20–50 μg) were separated by

SDS–PAGE, transferred onto PVDF membranes (Millipore), and probed with a primary antibody against target protein. The results were analyzed by Adobe Photoshop software and normalized to actin. For complex coimmunoprecipitation, cell extracts were prepared by using lysis buffer (50 mM Tris, pH 7.4, 150 mM NaCl, and 0.5% (vol/vol) Nonidet P-40, 1 mM EDTA) supplemented with a protease inhibitor cocktail (Roche). Lysates were incubated with the anti-flag (M2)-agarose or antibodycoupled beads for 3 h at 4°C.The immunoprecipitates were washed three times with the same buffer and subjected to immunoblot analysis.

**GST pull-down assay**

The fusion proteins of GST, GST-β-catenin, GST-β-catenin-ARM, GST-β-catenin-Δ634-663, GST-β-catenin-S33A and MBP-IRF3 were expressed in *E. coli B21* strain and purified according to standard protocols with pre-equilibrated GST beads. For GST pull-down assay, approximately 1 μg GST-fusion proteins mix with 20 μl pre-cleared agarose beads in 500 μl reaction medium, followed by adding 1 μg MBP-IRF3 and incubating at 4°C for 4 hours with gentle rotation. The precipitates were extensively washed three times and then eluted with 2× loading buffer, boil and then run SDS–PAGE gel.

**Generation of Flag-tagged IRF3, IRF3-ΔnDB, IRF3-ΔNLS and IRF3-5D overexpression lentivirus**

Flag-tagged IRF3, IRF3-ΔnDB, IRF3-ΔNLS and IRF3-5D DNA were cloned into pHAGE plasmid and lentivirus was packaged as previous described with little modification. Briefly HEK293FT cells in the 10 cm dish were transfected with 10 μg of Flag-tagged plasmids together with 10 μg of psPAX2 and 5 μg of pMD2G. 10 hours after transfection, the media were changed with fresh 10% FBS DMEM. Another 48 h later, the supernatant media were collected and centrifuged at 1,500 g for 10 min. The supernatant was filtered through a 0.22 mm membrane and stored at -80 °C. The specific primers for these plasmids construction are in shown as follows: **Supplementary Table 4.**

**Cell Proliferation Assay**

Cell proliferation was measured using the SynergyMx M5 (Molecular Devices). HCT116 or H1299 cells were plated at a density of 5,000 cells or 3,000 cells per well in 96-well culture plates and starved overnight. The serum stimulation was a time-decreased course, and plates were read at 96 hr. The number of cells could be represented by the different absorbance. For all statistical tests, $p<0.05$ with unpaired Student's test was considered statistically significant. In all the results, *$p<0.05$, **$p<0.01$, and ***$p<0.001$, NS denotes not statistically significant. Each sample was performed in triplicate and each experiment was repeated three times independently.

**Colony formation assay**

The HCT116 and SW620 cell lines were trypsinized and seeded into six-well plates with a density of 200 cells per well, and H1299 cells were seeded into six-well plates with a density of 100 cells per well. And then the cells were kept in an incubator at 37˚C and 5％ $CO_2$ . After seven days, the colonies were washed with 1× PBS, and stained with methyl violet. Finally, the methyl violet dye was washed off with PBS twice. The number of colonies was directly counted. Colony formation rate was calculated with the equation: relative colony-formation efficiency = (number of colonies/number of seeded cells)*100％.

**Tumor Formation Assay**

HCT116 or H1299 cells were infected with control plasmid, Flag-tagged IRF3, -IRF3-ΔnDB, -IRF3-ΔNLS and IRF3-5D mutations lentivirus, and selected by FCS for stable expression cells and cultured in culture medium for 2 weeks. Then HCT116 or H1299 cells were trypsinized into single cell

suspensions and resuspended in PBS. Approximately 2 × $10^6$ HCT116 cells and 5 × $10^6$ H1299 cells in 200 μl were injected into dorsal of nude mice. The formation of tumor was examined after 21 days.

**Generation and validation of IRF3-KO and Ctnnb1-KO Cells**

The IRF3 and Ctnnb1 knockout (KO) cells were constructed using the CRISPR/Cas9 gene-editing system. The CRISPR plasmid pEP-330x (Addgene) contains expression cassettes of Cas9 and puromycin resistant gene. The target sequences of gRNAs were designed using the MIT online tool (http://crispr.mit.edu/). To generate IRF3 KO cells, two gRNAs targeting the exons of IRF3 were designed and inserted into the pEP-330x vector by using the BpiI (Thermo) site, and then co-transfected into HCT116, SW620 and H1299 cells using Lipofectamine 3000 (Invitrogen) for 48 h. To generate Ctnnb1 KO cells, we designed two gRNAs to target the exons of Ctnnb1 and cloned into the pEP-330x vector, then the plasmids were co-transfected into HCT116 cells using Lipofectamine 3000 for 48 h. Then puromycin (Sigma-Aldrich) was used for selection in HCT116 (1 μg/ml), SW620 (2 μg/ml) and H1299 (3 μg/ml). To validate and pick out the knockout cell colonies, genomic DNA was extracted from the parental and knockout cells, and validated at DNA level by PCR and western blot. Primers for constructing CRISPR-CAS9 plasmids and genotype identification are listed in **Supplementary Table 5**.

**TOPflash luciferase activity assay**

HEK293T cells were co-transfected with a mixture of the indicated firefly luciferase reporter plasmid, the pRL-TK-Renilla-luciferase reporter assay plasmid, and any appropriate additional constructs for 18~24 hours. Total amounts of DNA were equalized with empty control vector. Luciferase activity was measured with the Dual-Luciferase Reporter Assay system (Promega) according to the manufacturer's instructions. Data were normalized for transfection efficiency by division of firefly luciferase activity by that of Renilla luciferase.

**Nuclear and cytoplasmic proteins extraction**

At least 5 × $10^6$ HCT116 or H1299 cells were used for nuclear and cytoplasmic proteins extraction by Nucleoprotein Extraction Kit (C500009-0050, Sangon Biotech) according to the manufacturer's instructions. GAPDH and histone H3 were used as cytoplasmic and nuclear loading controls respectively.

**The dosage of ICG-001**

HCT116 cells were treated with ICG-001 (50 μM) or DMSO in MTT assay. The antitumor effect (200 mg/kg, i.v.) was investigated in the HCT116 mouse xenograft model. The $IRF3^{fl/fl}$ and $IRF3^{fl/fl}$ $Villin^{cre}$ mice were treated orally with ICG-001 (300 mg/kg per day, once daily, six times per week) or vehicle, for the last 10 weeks of the AOM/DSS model.

**The dosage of G007-LK**

The $IRF3^{fl/fl}$ and $IRF3^{fl/fl}$ $Villin^{cre}$ mice were treated orally with G007-LK (50 mg/kg per day, once every three days) or vehicle, for the last 10 weeks of the AOM/DSS model.

**$Apc^{min/+}$ and $Apc^{min/+}IRF3^{-/-}$ mice antibiotic treatment**

Antibiotic treatment of mice was performed by supplementation of drinking water with ampicillin (1 mg/ml), gentamicin (1 mg/ml), metronidazole (1 mg/ml), neomycin (1 mg/ml), and vancomycin (0.5 mg/ml). For long-term antibiotic treatment, drinking water supplemented with antibiotics was continuously provided to mice from the age of one month until sacrifice at the age of five months.

**Protocol for Wnt3a-Conditioned Medium**

Wnt3a-conditioned medium is prepared following the guideline of ATCC website (https://www.atcc.org/products/all/CRL-2647.aspx#culturemethod) : Firstly, split the L-wnt3a cells

1:10 in 10 mL culture medium (without G418) in 10 cm tissue culture dishes and let the cells grow for 4 days (approximately to confluency); secondly, take off the medium to get the first batch of medium. Add 10 mL fresh culture medium and culture for another 3 days to get the second batch of medium. Finally, mix the first batch with second batch of medium (1:1), which is the wnt3a conditioned medium.

**Supplementary Table 2. List of 65 genes that only up-regulated in the tumor tissue in "KO" group**

Tnfsf4, Csf3, Pax2, Wnt7a, Hrg, Ppbp, Trp63, Krt4, Cdkn2a, Tbx1, Wnt7b, Uts2r, Foxn1, Mycn, Gm13283, Npy5r, Nppb, Rtkn2, Aldh3a1, Fgf18, Msx1, Tfap2a, Cxcl10, Nppc, Serpine1, Tnfrsf9, Irs1, Tnfsf9, Pdgfd, Dmrta2, Ascl2, Il1a, Apln, Thpo, Dusp22, Ptgs2, Prkd1, Magi2, E2f7, Pla2g2d, Ccl2, Ngfr, Clcf1, Ang4, Bok, Hmga2, Btc, Egln3, Nfatc2, Jag2, Mtbp, Etv4, Fzd6, Lif, Aspm, Tnfrsf4, Brca2, Amica1, Mfge8, Myc, Rps6ka2, Hmgn1, Hgf, Edn1, Pola1.

**Supplementary Table 3. List of qPCR primers**

| | | |
|---|---|---|
| Mouse Actin | Forward | AACAGTCCGCCTAGAAGCAC |
| | Reverse | CGTTGACATCCGTAAAGACC |
| Mouse IL6 | Forward | AGTTGCCTTCTTGGGACTGA |
| | Reverse | TCCACGATTTCCCAGAGAAC |
| Mouse TNFα | Forward | CTGGGACAGTGACCTGGCT |
| | Reverse | GCACCTCAGGGAAGAGTCTG |
| Mouse CXCL1 | Forward | CGCTTCTCTGTGCAGCGCTGCTGCT |
| | Reverse | AAGCCTCGCGACCATTCTTGAGTG |
| Mouse CCl20 | Forward | AACTGGGTGAAAAGGGCTGT |
| | Reverse | GTCCAATTCCATCCCAAAAA |
| Mouse IL1β | Forward | CCAAAAGATGAAGGGCTGCT |
| | Reverse | ACAGAGGATGGGCTCTTCT |
| Mouse Myc | Forward | GATGGAGATGAGCCCGACT |
| | Reverse | CCTAGTGCTGCATGAGGAGAC |
| Mouse CyclinD1 | Forward | CTGCAAATGGAACTGCTTCTGGTGA |
| | Reverse | AGCAGGAGAGGAAGTTGTTGGGGCT |
| Mouse Axin2 | Forward | ACCAGGATGGTGCATACCTCT |
| | Reverse | CCCATTACA AGCAAACCAGAAGT |
| Mouse Lef1 | Forward | TGTTTATCCCATCACGGGTGG |
| | Reverse | CATGGAAGTGTCGCCTGACAG |
| Mouse TCF1 | Forward | AGCTTTCTCCACTCTACGAACA |
| | Reverse | AATCCAGAGAGATCGGGGGTC |
| Mouse Lgr5 | Forward | CTCCACACTTCGGACTCAACAG |
| | Reverse | AACCAAGCTAAATGCACCGAAT |
| Mouse Ascl2 | Forward | GGAGCTGCTTGACTTTTCCAG |
| | Reverse | CATTTGGTCAGGCTGCACTAG |
| Mouse CD44v6 | Forward | AGAAAATTGGACCCCGGAAC |

| | | |
|---|---|---|
| | Reverse | GAATGACTCTGTGTGGTGGC |
| Mouse Olfm4 | Forward | CAGCCACTTTCCAATTTCACTG |
| | Reverse | GCTGGACATACTCCTTCACCTTA |
| Mouse CTCG | Forward | GGACACCTAAAATCGCCAAGC |
| | Reverse | ACTTAGCCCTGTATGTCTTCACA |
| Mouse Cyr61 | Forward | TAAGGTCTGCGCTAAACAACTC |
| | Reverse | CAGATCCCTTTCAGAGCGGT |
| Human GADPH | Forward | ATTCCACCCATGGCAAATTC |
| | Reverse | GGATCTCGCTCCTGCAAGATG |
| Human Myc | Forward | TTCGGGTAGTGGAAAACCAG |
| | Reverse | CAGCAGCTCGAATTTCTTCC |
| Human CyclinD1 | Forward | TGGAGCCCGTGAAAAAGAGC |
| | Reverse | TCTCCTTCATCTTAGAGGCCAC |
| Human Axin2 | Forward | CTGGCTCCAGAAGATCACAAAG |
| | Reverse | ATCTCCTCAAACACCGCTCCA |
| Human Tcf1 | Forward | CTGGCTTCTACTCCCTGACCT |
| | Reverse | ACCAGAACCTAGCATCAAGGA |
| Human Lef1 | Forward | AGAACACCCCGATGACGGA |
| | Reverse | GGCATCATTATGTACCCGGAAT |
| Human Lgr5 | Forward | CTTCCAACCTCAGCGTCTTC |
| | Reverse | TTTCCCGCAAGACGTAACTC |
| Human Olfm4 | Forward | ACCTTTCCCGTGGACAGAGT |
| | Reverse | TGGACATATTCCCTCACTTTGGA |
| Human CD133 | Forward | CTGGGGCTGCTGTTTATTATTCTG |
| | Reverse | ACGCCTTGTCCTTGGTAGTGTTG |

**Supplementary Table 4. List of primers for pHAGE plasmids construction**

| | | |
|---|---|---|
| pHAGE-IRF3 | Forward | GACGACGATGACAAGCTCGAGATGGGAACCCCAAAGCCACGGATC |
| | Reverse | CAGGCCTGGAAGATTCCGAAATCCTCGAGCTTGTCATCGTCGTC |
| pHAGE-IRF3-ΔnDB | Forward | GACGACGATGACAAGCTCGAGGATTTCGGAATCTTCCAGGCCTG |
| | Reverse | CAGGCCTGGAAGATTCCGAAATCCTCGAGCTTGTCATCGTCGTC |
| pHAGE-IRF3-ΔNLS-1 | Forward | GCCAGACCTGCCAACCTGGAACGGGAATTTCCGCTCTGCCCTCAAC |
| | Reverse | GAGGGCAGAGCGGAAATTCCCGTTCCAGGTTGGCAGGTCTGGC |
| pHAGE-IRF3-ΔNLS-2 | Forward | GAATTTCCGCTCTGCCCTCAACCTCCAAGAAGGGTTGCGTTTAGCAG |
| | Reverse | CTGCTAAACGCAACCCTTCTTGGAGGTTGAGGGCAGAGCGGAAATTC |

| | | |
|---|---|---|
| pHAGE-IRF3-5D-1 | Forward | GTGGACCTGCACATTGACAACGACCACCC ACTCTC |
| | Reverse | GAGAGTGGGTGGTCGTTGTCAATGTGCAG GTCCAC |
| pHAGE-IRF3-5D-2 | Forward | CAACGACCACCCACTCGACCTCGACGACG ACCAGTAC |
| | Reverse | GTACTGGTCGTCGTCGAGGTCGAGTGGGTG GTCGTTG |

**Supplementary Table 5. List of primers for constructing CRISPR-Cas9 plasmids**

| | | |
|---|---|---|
| Human IRF3-1# | Guide | ACCGGAAGGATGTGCCATGGATA |
| | Insert | AACTATCCATGGCACATCCTTCC |
| Human IRF3-2# | Guide | ACCGAAAGCCGGTTAGTACGCAA |
| | Insert | AACTTGCGTACTAACCGGCTTTC |
| Human Ctnnb1-1# | Guide | ACCGAATAGTGTTTTCCCCTCCC |
| | Insert | AACGGGAGGGGAAAACACTATTC |
| Human Ctnnb1-2# | Guide | ACCGGATGACTGCTTCTGGAGCC |
| | Insert | AACGGCTCCAGAAGCAGTCATCC |
| Human IRF3 cas9 identification primer | Forward | GCTTACACGTGAATCGATTCTG |
| | Reverse | CTTGGTGTCTAATCCCGTGC |
| Human Ctnnb1 cas9 identification primer | Forward | GTTATCCAAAGGGGATCTGTG |
| | Reverse | CAGAGTCCAGGTAAGACTGTTGC |

**Supplementary Table 6. List of antibody information**

| **Antibodies** | **SOURCE** | **IDENTIFIRS** |
|---|---|---|
| IRF3 (rabbit) | Proteintech | 11312-1-AP |
| LEF1 (rabbit) | Proteintech | 14972-1-AP |
| Phospho-IRF3 (Ser386) (rabbit) | Cell Signaling Technology | #37829 |
| β-Catenin (D10A8) (rabbit) | Cell Signaling Technology | #8480 |
| Non-phospho(Active) β-Catenin (Ser33/37/Thr41) (rabbit) | Cell Signaling Technology | #8814 |
| Phospho-β-Catenin (Ser33/37/Thr41) (rabbit) | Cell Signaling Technology | #9561 |
| β-Catenin (mouse) | Cell Signaling Technology | #2677 |
| Phospho-Stat3 (rabbit) | Cell Signaling Technology | #9145 |
| Akt Antibody (rabbit) | Cell Signaling Technology | #9272 |
| Phospho-Akt (Ser473) (rabbit) | Cell Signaling Technology | #4060 |
| Phospho-JNK (rabbit) | Cell Signaling Technology | #4668 |
| JNK Antibody (rabbit) | Cell Signaling Technology | #9252 |
| Phospho-Erk1/2 (rabbit) | Cell Signaling Technology | #4370 |
| Erk1/2 (rabbit) | Cell Signaling Technology | #4695 |
| Phospho-p38 (rabbit) | Cell Signaling Technology | #4511 |
| p38 Antibody(rabbit) | Cell Signaling Technology | #9212 |

| | | |
|---|---|---|
| Cyclin D1 Antibody (rabbit) | Cell Signaling Technology | #2978 |
| c-Myc Antibody (rabbit) | Cell Signaling Technology | #5605 |
| Histone H3 Antibody (rabbit) | Cell Signaling Technology | #4499 |
| TCF1/TCF7 Antibody (rabbit) | Cell Signaling Technology | #2203 |
| STING (D2P2F) | Cell Signaling Technology | #13647 |
| MAVS (D5A9E) | Cell Signaling Technology | #24930 |
| β-Tubulin Antibody | Cell Signaling Technology | #2148 |
| Lysozyme antibody (rabbit) | Abcam | ab108508 |
| HA-Tag antibody (rabbit) | Cell Signaling Technology | #3724 |
| HA Tag antibody (mouse) | Abmart | ABCA0250882 |
| Anti-flag antibody (rabbit) | DiagBio | db7002 |
| Flag Tag antibody (mouse) | Abmart | ABCA2261255 |
| His-Tag (10E2) | Abmart | M30111 |
| GAPDH antibody (rabbit) | Cell Signaling Technology | #5174 |
| GST antibody (mouse) | HUABIO | EM80701 |
| MBP antibody (rabbit) | HUABIO | ER1912-82 |

## Supplementary References


1. Iris D Nagtegaal, et al., *Tumours of the colon and rectum.* WHO Classification of Tumours Editorial Board, 2019. **The WHO classification of digestive system tumours. 5th edition**: p. 158-211.
